# Unusually Large Effects of Charge‐assisted C−H⋅⋅⋅F Hydrogen Bonds to Anionic Fluorine in Organic Solvents: Computational Study of ^19^F NMR Shifts versus Thermochemistry

**DOI:** 10.1002/open.202200146

**Published:** 2022-08-19

**Authors:** Martin Kaupp, Caspar J. Schattenberg, Robert Müller, Marc Reimann

**Affiliations:** ^1^ Technische Universität Berlin Institut für Chemie, Theoretische Chemie/Quantenchemie Sekr. C7, Strasse des 17. Juni 135 10623 Berlin Germany

**Keywords:** NMR chemical shifts, solvation effects, hydrogen bonding, density functional theory, solvation free energy

## Abstract

A comparison of computed ^19^F NMR chemical shifts and experiment provides evidence for large specific solvent effects for fluoride‐type anions interacting with the σ*(C−H) orbitals in organic solvents like MeCN or CH_2_Cl_2_. We show this for systems ranging from the fluoride ion and the bifluoride ion [FHF]^−^ to polyhalogen anions [ClF_x_]^−^. Discrepancies between computed and experimental shifts when using continuum solvent models like COSMO or force‐field‐based descriptions like the 3D‐RISM‐SCF model show specific orbital interactions that require a quantum‐mechanical treatment of the solvent molecules. This is confirmed by orbital analyses of the shielding constants, while less negatively charged fluorine atoms (e. g., in [EF_4_]^−^) do not require such quantum‐mechanical treatments to achieve reasonable accuracy. The larger ^19^F solvent shift of fluoride in MeCN compared to water is due to the larger coordination number in the former. These observations are due to unusually strong charge‐assisted C−H⋅⋅⋅F^−^ hydrogen bonds, which manifest beyond some threshold negative natural charge on fluorine of ca. < −0.6 e. The interactions are accompanied by sizable free energies of solvation, in the order F^−^≫[FHF]^−^>[ClF_2_]^−^>[ClF_4_]^−^. COSMO‐RS solvation free energies tend to moderately underestimate those from the micro‐solvated cluster treatment. Red‐shifted and intense vibrational C−H stretching bands, potentially accessible in bulk solution, are further spectroscopic finger prints.

## Graphical abstract

**Strong charge‐assisted** CH⋅⋅⋅F hydrogen bonds to negatively charged fluorine atoms in organic solvents like acetonitrile lead to strong solvation and can have surprisingly large effects on the ^19^F NMR shifts of the solute anions. A proper quantum‐chemical treatment requires the use of microsolvated cluster models.

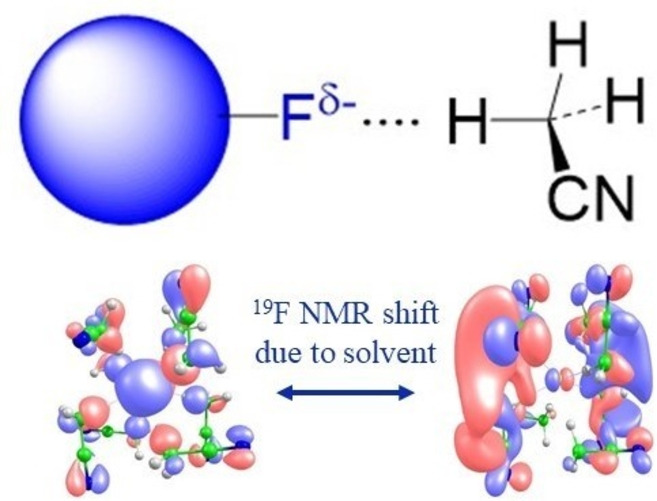

## Introduction

CH bonds acting as hydrogen‐bond donors have a long and varied history going back to a 1937 suggestion by Glasstone to explain the formation of mixtures of CX_3_H (X=Cl, Br, I) with acetone or quinoline.^[1]^ From the controversial early history of such suggestions, we mention here only the X‐ray diffraction studies of short CH⋅⋅⋅O distances in crystals by Suton^[2]^ and first definitive IR experiments by Allerhand and Schleyer.^[3]^ Meanwhile the existence of such interactions is very well established, with too many facets and applications to be mentioned here in detail. They even form the central basis for a book on weak hydrogen bonds.^[4]^ The latter implies that usually such interactions tend to be weaker than regular hydrogen bonds with more electronegative donor atoms like N, O, or F. And indeed, often CH⋅⋅⋅O hydrogen bonds tend to exhibit blue‐shifted CH vibrations with reduced intensities and have been (improperly) termed “improper hydrogen bonds”, as the charge‐transfer component is small, and electrostatic and rehybridization effects can lead to shortened CH bonds with enhanced force constants and reduced bond dipoles.[^5^, ^6^]

However, the strength of CH⋅⋅⋅X hydrogen bonds can be enhanced by various factors: a) a net positive charge on the donor in cationic species, for example by protonation, metal coordination or involvement in an ammonium‐ion framework;^[7]^ b) employing sp^2^ or sp hybridized carbon donors, which increases their electronegativity;^[8]^ c) a more gradual increase of the electronegativity and donor strength by attaching electronegative substituents to the carbon atom (this explains the predominant early observation of such interactions for haloforms); and d) a negative charge on the hydrogen‐bond acceptor.[^9^, ^10^, ^11^] All of these aspects have been utilized, for example, in the design of supramolecular anion receptors,^[12]^ including those for halide ions, or, for example, in catalysis.^[13]^ The term “charge‐assisted hydrogen bonding” has been used in this context.^[14]^

While these types of studies of specific anion receptors benefit from well‐defined structural arrangements, the CH bonds of many common solvents also should be expected to form rather strong CH⋅⋅⋅X hydrogen bonds with anionic solutes. This is supported by a number of recent computational studies at various levels.[^9^, ^10^, ^11^] While little experimental solution data is available, these computational studies indeed suggest strong CH⋅⋅⋅X interactions to contribute to the microsolvation of anions in many common organic solvents. This certainly should hold for aliphatic CH groups not only in the haloforms (which have been used in previous computational studies^[9]^) but also in solvents like acetonitrile (MeCN) or dichloromethane (DCM), which all bear electronegative substituents on the donor carbon atom. It should also hold for the more electronegative aromatic CH groups as in benzene or toluene, even in the absence of electronegative substituents,^[15]^ but enhanced in their presence.^[16]^

If we discard small, multiply charged anions,^[10a]^ which are unlikely to dissolve in organic solvents, fluoride‐like species may be expected to exhibit the largest CH⋅⋅⋅X solvent‐solute interactions,^[9]^ as fluoride concentrates negative charge density on the smallest conceivable volume. In a recent combined experimental and computational study^[17]^ of polyhalogen anions [EF_x_]^−^ (E=Cl, Br, I; X=2, 4, 6) we found that using suitable DFT approaches and continuum solvent models allowed us to reproduce well the ^19^F NMR shifts with X=4, 6 to within about 10–15 ppm but not with X=2. In the latter case our computations underestimated the shifts systematically by about 50–60 ppm at our best DFT level used (with the LH12ct‐SsifPW92 local hybrid functional^[18]^ and large basis sets). As relativistic effects were found to be small, and such discrepancies are clearly outside the expected accuracy of functional and basis set,[^19^, ^20^] we suspected that specific solvent interactions in the experimentally used MeCN, that are not covered adequately by the standard COSMO solvent model^[21]^ used, are responsible for the differences. We will show this to be the case below by using microsolvated cluster models. The differences between X=2 versus X=4, 6 in these systems intrigued us, as they suggested that the negative charge on fluorine may determine how well or how poorly standard solvent models may reproduce these effects. Similar specific solvent interactions can be inferred from recent ab initio molecular dynamics studies of the bifluoride ion [FHF]^−^ in deuterated dichloromethane (CD_2_Cl_2_).^[22]^ Here an explicit quantum‐mechanical treatment of solvent molecules was found as well to be necessary to reproduce the experimental ^19^F NMR shifts in solution, even though the main focus of that work was on the (a‐)symmetry of hydrogen bonding within the anion.

Properties like NMR shifts are important probes of molecular interactions, and the need to include solute‐solvent interactions quantum‐mechanically in some but not in other cases signals a more general challenge for computational studies in that field. Consider, for example, a chemical reaction where the anionic character on a fluorine atom (or on some other very electronegative atom) varies for different intermediates or transition states. Then standard computational treatments using continuum solvent models will certainly not be adequate, and an appropriate treatment of microsolvation becomes mandatory. As we will see below, a force‐field based treatment of the solvent, as in QM/MM simulations or related approaches, is also insufficient.

To obtain more insight, we use here microsolvated model clusters for anionic fluorine species and select MeCN as organic solvent and CH‐bond donor, as it is often used to dissolve such species (see Ref. [11] for a recent computational study of interactions between one acetonitrile molecule and chloride). In addition to the anions [ClF_x_]^−^ (X=2, 4) that were part of the abovementioned study,^[17]^ we will evaluate such effects for the free fluoride ion, and for the bifluoride ion. We will concentrate on MeCN, to avoid complications due to halogen bonding, as is possible for DCM. For the fluoride ion we will compare also to aqueous solution, as experimental evidence suggests a larger deshielding solvent effect on the ^19^F shift in organic solvents such as MeCN compared to water,^[23]^ which seems interesting to understand.

## Computational Details

Initial estimates of the average number of solvent molecules expected around a given species were obtained using 3D‐RISM‐SCF calculations,^[24]^ where the solute is treated at the BP86‐D3(BJ)/TZ2P level[^25^, ^26^, ^27^] and the solvent at the 3D‐RISM level based on OPLS force field parameters, either using a united atom (UA) approach^[28]^ or the parameters obtained from the LigParGen web server[^29^, ^30^] employing 1.14*CM1A‐LBCC^[31]^ charges and the all‐atoms (AA) approach. All these computations used a modified version of our recent 3D‐RISM‐SCF implementation^[32]^ in the ADF engine^[33]^ of the AMS program package^[34]^ – a code based on Slater‐type‐orbital basis sets. These modifications are part of the 2022.1 release. In all calculations, the 3D‐RISM equations were solved on a Cartesian grid with 128 points in each direction and a spacing of 0.25 Å using the KH closure^[35]^ and solvent susceptibility functions obtained from DRISM calculations^[36]^ employing the hypernetted chain (HNC) closure.^[37]^ The Lennard‐Jones parameters of the solute atoms were taken from Ref. [38], using special parameters for the H in FHF^−^ (σ=1.0 Å, ϵ=0.056 kcal/mol). The electrostatic potential obtained from the fitted electron density of the solute at the given DFT level has been used.^[32]^ The estimated average coordination number was obtained by integrating the pair distribution function – either spherically averaged around a fluorine atom or around the molecular center of mass – up to the first minimum.

The computed average solvation numbers provided the basis for fast meta‐dynamics runs using the GFN2‐xTB tight‐binding approach^[39]^ as implemented in the CREST tool of the xtb program.^[40]^ Optimized structures from the latter simulations were used as starting points for DFT structure optimizations of clusters of the solute with varying numbers of solvent molecules. These optimizations were done within a COSMO solvent environment (ϵ=35.688 for MeCN, ϵ=78.355 for water) at MARIJ‐BP86‐D3(BJ)/def2‐TZVPP^[41]^ level (MARIJ stands for “multipole‐accelerated resolution of the identity”), using the Turbomole program,[^42^, ^43^] version 7.5.1 and newer.

Free energies of solvation were computed from the microsolvated cluster energies following a cluster cycle^[44]^ corresponding to the reaction [X^−^(MeCN)_n_]_solv_⇌X^−^(g)+[(MeCN)_n_]_solv_ (and analogously for F^−^(H_2_O)_n_). Contributions to the free energies were obtained from the optimized clusters by including their vibrational contributions at the same level plus additional solvation contributions at COSMO‐RS(MeCN) level for X^−^(MeCN)_n_ as well as for (MeCN)_n_.^[45]^ To this end, additional single‐point calculations at the COSMO and gas‐phase optimized structures were carried out. These calculations were performed at MARIJ‐BP86 level (omitting the D3(BJ) corrections) with COSMO, setting an infinite permittivity and using the refined COSMO cavity construction algorithm (keyword $cosmo_isorad),^[45b]^ as well as in the gas phase, employing def2‐TZVPD^[41a]^ basis sets for all atoms. Based on these single‐point calculations, subsequent COSMO‐RS computations to obtain ΔG_solv_ used the COSMOtherm program, version C30_1201, and a BP‐TZVPD‐FINE level parameterization (BP_TZVPD_FINE_HB2012_C30_1201). Electronic energy contributions were refined by single‐point energy calculations at the DLPNO‐SCS‐MP2/aug‐cc‐pVTZ[^46^, ^47^] level and, where computationally feasible for smaller clusters, at the DLPNO‐CCSD(T)‐F12/cc‐pVTZ‐F12 level as reference.[^48^, ^49^] These calculations used the ORCA program, version 4.2.1.^[50]^ For comparison, electronic energies have also been computed by single‐point energy calculations at the ωB97M‐V/def2‐TZVPP^[51]^ DFT level. Furthermore, free energies of solvation without the presence of explicit solvent molecules have been computed directly with COSMO‐RS at MARIJ‐BP86/def2‐TZVPD level for comparison, using the settings given above. Free energies were computed at standard conditions, i. e. 298.15 K and 0.1 MPa, and standard‐state corrections were computed as SScorr=−RTln(V_m_); V_m_=24.46 mol l^−1^.

Using the clusters optimized at MARIJ‐BP86‐D3(BJ)/def2‐TZVPP/COSMO level, calculations of ^19^F shieldings were done with a number of different DFT functionals that have recently been established to perform well for the ^19^F shielding and shift subset (47 nuclei) of the large coupled‐cluster‐based NS372 benchmark.^[20]^ That is, we used the three local hybrid functionals cLH12ct‐SsirPW92,^[18]^ LH12ct‐SsifPW92,^[18]^ and LH20t^[52]^ including their current‐density response[^53^, ^54^] within Dobson's scheme^[55]^ (denoted cLH12ct‐SsirPW92, cLH12ct‐SsifPW92 and cLH20t) and with gauge‐including atomic orbitals (GIAOs^[56]^) as implemented in the Turbomole code, version 7.6. pcSseg‐3 basis sets were employed for all atoms, and the computations used COSMO (MeCN, ϵ=35.688; H_2_O, ϵ=8.93), as well as the MARIJ approach for the Coulomb contribution (using “universal” auxiliary basis sets^[57]^). We will preferably focus on results obtained with the cLH12ct‐SsirPW92 local hybrid functional. For free F^−^ and for HF, it provides ^19^F shielding constants that agree with high‐level CCSD(T)/pcSseg‐3^[58]^ results to within about 1 ppm, and for LiF to within ca. 3 ppm.^[20]^ Its mean absolute error for the full ^19^F subset of the recent NS372 benchmark is 9.7 ppm.^[20]^ This should be compared to 5.2 ppm for MP2 and to 9.0 ppm for the top‐performing[^20^, ^59^] double hybrid DSD‐PBEP86,^[60]^ which both require substantially higher computational effort and indeed exhibit somewhat larger deviations from the benchmark data for HF (in the latter case also for LiF). For comparison we also used the BHLYP global hybrid functional^[61]^ that has previously been used often for ^19^F NMR parameter computations^[62]^ but does not perform as well as the three local hybrids.[^19^, ^20^, ^53^, ^54^] Computations without explicit microsolvation using COSMO or the self‐consistent version of COSMO‐RS, D‐COSMO‐RS,^[63]^ were also done for comparison at the otherwise identical levels. Additional computations of ^19^F shieldings using 3D‐RISM‐SCF as solvent model without explicit solvent molecules used ADF/AMS at BHLYP/QZ4P‐J//BP86‐D3(BJ)/TZ2P level.

To transform computed ^19^F absolute shielding constants σ into chemical shifts δ requires a value for the absolute shielding σ_ref_ of the reference standard used experimentally. For ^19^F NMR this is nontrivial, as a direct computation of the absolute shielding constant of neat liquid CFCl_3_ is required. In this work we decided to indeed use reference shieldings for CFCl_3_ computed directly at the given level, using structures optimized at the MARIJ‐BP86‐D3(BJ)/def2‐TZVPP level with COSMO (ϵ=2.315 for liquid CFCl_3_ ^[64]^) to model the liquid environment. Shielding computations also used COSMO(CFCl_3_). The obtained reference values are: 186.3 ppm (cLH12ct‐SsirPW92), 189.5 ppm (cLH12ct‐SsifPW92), 184.6 ppm (cLH20 t), and 187.2 ppm (BHLYP).

We have carefully considered but ultimately not applied an alternative scheme for obtaining a shielding for liquid CFCl_3_ indirectly via the secondary standard of gaseous dilute HF at 300 K that we want to share here for scientists interested in computing ^19^F NMR shifts in other contexts: the relative shift of HF(g, 300 K) with respect to neat liquid CFCl_3_ can be inferred to be 217.02 ppm from a relative shift of HF(g, 300 K) against SiF_4_(g, 300 K) of 46.85±0.35 ppm,^[65]^ and a more recent relative shift of SiF_4_(g, 300 K) versus CFCl_3_(l, 300 K) of 170.17 ppm.^[66]^ For the absolute shielding of HF(g, 300 K) we use a nonrelativistic value of 409.7 ppm computed from an equilibrium shielding of 419.384 ppm obtained at CCSDTQ/CBS level (with corrections for quintuple excitations) and 300 K rovibrational corrections at CCSD(T)/CBS level of −9.677 ppm.^[67]^ Using this shielding value provides us with a reference shielding value of 192.7 ppm for neat liquid CFCl_3_. We assume that this is an appropriate reference for shielding computations that neglect relativistic effects, as we expect computed relativistic effects of ca. +4.6 ppm^[67]^ to be essentially atomic in nature (a so‐called heavy‐atom effect on the heavy‐atom shielding, HAHA^[68]^) and to therefore cancel out by taking the experimental shift of HF(g) vs. CFCl_3_(l) into account to obtain our reference shielding. When relativistic corrections would be included in the actual shielding computations, the reference value would have to be increased accordingly. Note that this alternative reference shielding value is a few ppm larger than the directly computed ones (see above), and it would thus lead to slightly lower relative shifts. We note furthermore in passing that all σ_ref_ values discussed here are much lower than the MP2‐based gas‐phase value of 217.9 ppm used in the MP2 computations of fluoride solvent shifts in Ref. [23], explaining our more negative shifts for free fluoride compared to the −260 ppm given in that work.

Natural population analyses (NPA) and natural bond orbital analyses (NBO)^[69]^ for the clusters F^−^(H_2_O)_6_ and F^−^(MeCN)_8_ were carried out at the BP86‐D3(BJ)/def2‐TZVPP level using the NBO3.1 routines available in the Gaussian16 program, revision A.03.^[70]^ MO‐based analyses of the shielding constants computed at GIAO‐BP86/pcSseg‐2//MARIJ‐BP86‐D3(BJ)/def2‐TZVPP level with Turbomole were carried out by interfacing to the in‐house MAG code.^[71]^ Alternative analyses using localized MOs at IGLO‐level (individual gauges for localized orbitals^[72]^) were not as informative, and we will thus concentrate on the GIAO‐based canonical MO analyses. The Turbomole MOs have been generated with convergence criteria scfconv 10^−9^, convergence of the density matrix to 10^−7^, and gridsize m5 (internal settings). Harmonic vibrational spectra for cluster models were computed using the NumForce subroutine at MARIJ/BP86‐D3(BJ)/def2‐TZVPP/COSMO level with displacements of 0.02 a.u. Spectra are obtained with Gaussian broadening and a full width at half maximum of 4 cm^−1^ using the Gallier tool included in Turbomole.

## Results

### Fluoride in acetonitrile compared to water

**Figure 1:**
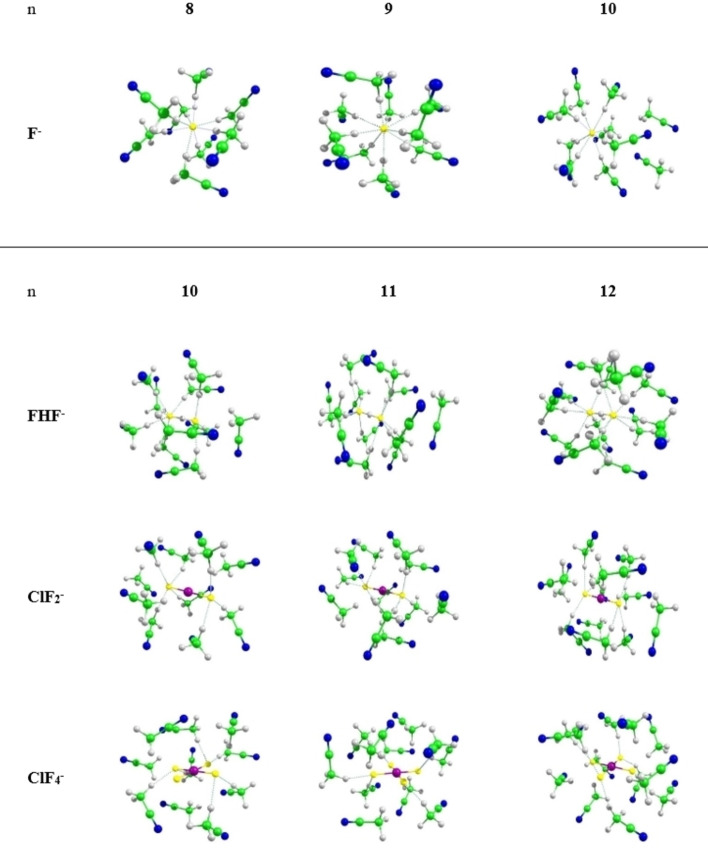
Examples of microsolvated clusters X^−^(MeCN)_n_ with relevant sizes (structures optimized at COSMO‐MARIJ/BP86‐D3(BJ)/def2‐TZVPP level).

We start with the fluoride ion as the simplest solute. For F^−^ in MeCN our 3D‐RISM‐SCF computations suggest an average coordination by 9–10 MeCN methyl groups in the first solvation shell for both UA and AA approaches. All‐atom calculations show that a bit more than 1 in 3 of the attached H atoms is in close contact with the fluoride ion (using a maximum F⋅⋅⋅H distance of 2.7 Å), suggesting each methyl group to contribute one CH bond oriented towards the fluoride (see Figures S1, S2 in Supporting Information for the computed radial distribution functions). This rather large coordination number is indeed confirmed by the GFN2‐xTB MD simulations and preoptimizations, which show binding of up to eight or nine solvent molecules in the first shell. DFT‐ and DFT‐COSMO‐optimized clusters tend to retain this picture. The F^‐^(MeCN)_9_ cluster has all nine MeCN molecules contributing to the coordination (Figure 1). For n=10, 11 we see only eight molecules coordinating directly to fluoride, with 3–4 somewhat shorter CH⋅⋅⋅F contacts of around 1.98 Å and the others somewhat longer, 2.04 Å (see Figure 1 and Table S1; for n=9 a somewhat different arrangement with overall longer distances is found).

**Figure 2:**
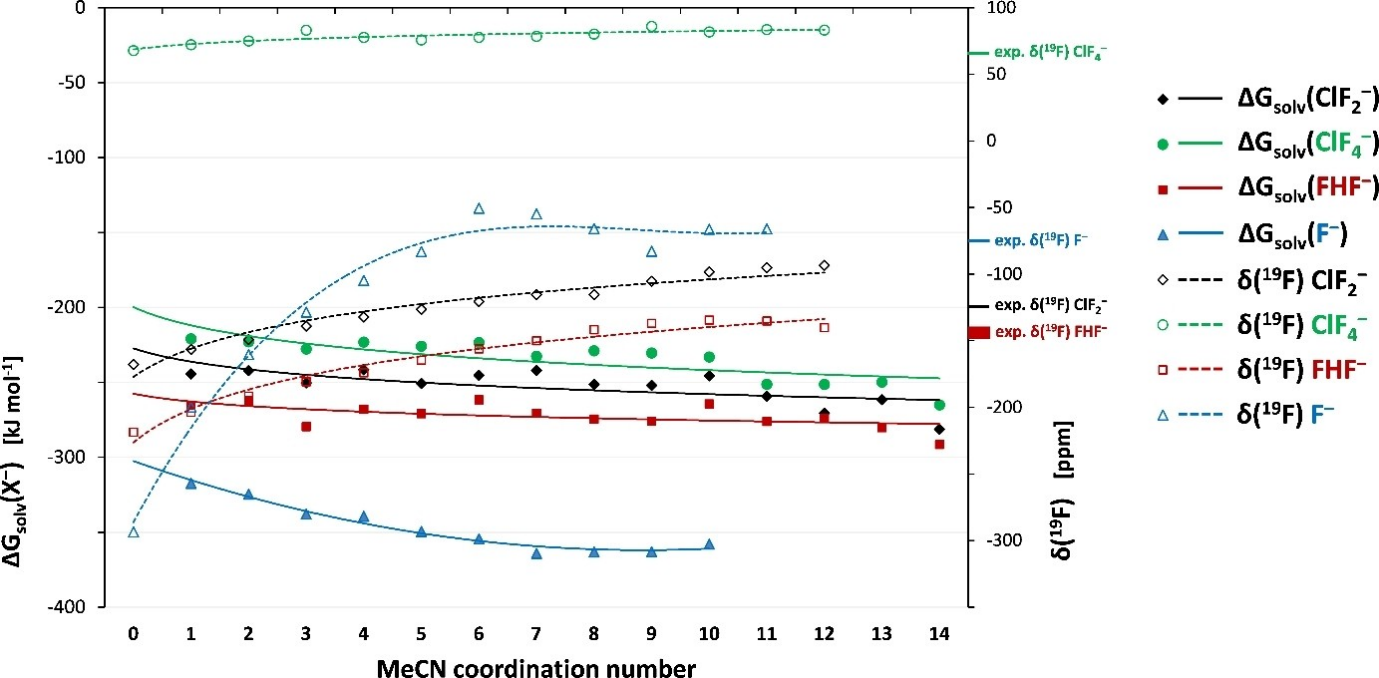
A comparison of computed solvation free energies ΔG_solv_ (DLPNO‐SCS‐MP2/aug‐cc‐pVTZ/COSMO‐RS//MARIJ‐BP86‐D3(BJ)/def2‐TZVPP[/COSMO(MeCN)] level using a cluster cycle) and ^19^F NMR chemical shifts (GIAO‐cLH12ct‐SsirPW92/pcSseg‐3/COSMO(MeCN)//MARIJ‐BP86‐D3(BJ)/def2‐TZVPP/COSMO(MeCN) level) of microsolvated anions in MeCN as a function of cluster size. See Supporting Information Table S2 for thermochemical data, Table S4 for NMR shifts, and Table S3 for comparative thermochemical data using a monomer cycle. Experimental shifts and shift ranges are shown at the right‐hand y‐axis.

The exergonicity of the free binding energies of the solvent molecules in the F^−^(MeCN)_n_ clusters increases very quickly for smaller n and then more slowly (Figure 2 and Table S2). The curve dips at around n=7–9, confirming completion of the first solvation shell at around this number. Note that the embedding of both the clusters and the cluster of MeCN molecules without fluoride by the COSMO‐RS solvent model is required to obtain these qualitatively correct curves, and it is mandatory to use a “cluster cycle” (see Computational Details). If one uses a “monomer cycle” according to the reaction [X^−^(MeCN)_n_]_solv_⇌X^−^(g)+n[(MeCN)]_solv_ rather than the cluster cycle, the most negative free energies (Table S3) are obtained for smaller n (around n=5 for F^−^), and the curves then bend up towards zero for larger n values. This incorrect behavior, which is shown in Figures S3, S4 (without and with COSMO‐RS embedding; see also Table S3) in Supporting Information and is similar to curves obtained for microsolvation of anions by CHF_3_,^[9]^ is an artefact of an exaggerated translational entropy for the monomer treatment. The even slightly larger coordination number given by 3D‐RISM‐SCF might be attributed to the somewhat larger CH⋅⋅⋅F bond length (maximum of the RDF, Figure S1). What is also noticeable is the rather steep negative slope from n=1 to n=2, which is consistent with the steep positive slope in that area for the ^19^F shift changes (Figure 2). A direct computation of the solvation free energy of F^−^ in MeCN with COSMO‐RS, without explicit solvent molecules, gives −375.2 kJ mol^−1^. This is somewhat below the expected asymptote of the cluster‐based free‐energy curve in Figure 2, and it agrees well with an experimental solvation free energy range of −383 kJ mol^−1^ to −389 kJ mol^−1^ for F^−^ in MeCN obtained from the known range in aqueous solution (−454 kJ mol^−1[73]^ to −460 kJ mol^−1[74]^) and the standard Gibbs free energy for the transfer of fluoride from water to MeCN (+71 kJ mol^−1^ at 298 K).^[75]^ Given the uncertainties involved in the explicit calculations on the microsolvated cluster models (e. g., neglected dynamical averaging, see below, or configurational entropies) as well as in the parametrization of COSMO‐RS, this may be considered close agreement. We note in passing that DLPNO‐CCSD(T)‐F12 gives about 13 kJ mol^−1^ more negative solvation free energies for a given n than the DLPNO‐MP2 values used to draw the curves in Figure 2 (compare Figure S5 in Supporting Information for electronic binding energies; BP86‐D3(BJ) and ωB97X‐D give still larger binding energies; see also Table S2), bringing the microsolvated‐cluster value to around −365 kJ mol^−1^, into even closer agreement with the implicit COSMO‐RS value and experiment. We note in passing that only for fluoride, the COSMO‐RS value is more negative than the (COSMO‐RS‐embedded) microsolvated cluster value, while this reverts for the other anions discussed below (see Figure S6).

The experimental ^19^F NMR shifts of fluoride in different solvents have been studied by Christe and coworkers (based on dissolving tetramethylammonium fluoride),^[76]^ and by others,^[77]^ and have been summarized and compared to MP2 calculations of fluoride with one coordinated solvent molecule (and up to six coordinated molecules for water) in Ref. [23]. Interestingly, the measured ^19^F shifts of fluoride in organic solvents are larger (less negative) than those in water or in alcoholic solvents (e. g. −74 ppm in MeCN, −109 ppm in DCM, −119 ppm in water, and −149 ppm in MeOH). That is, the deshielding solvent contribution in MeCN is particularly large. It was pointed out that these solvent shifts do not correlate with the binding energies of the corresponding solvent molecules to fluoride.^[23]^ The MP2 calculations of the monocoordinated clusters reproduce the trends but of course underestimate the solvent shifts. Our computed shifts for n=8 (−65.9 ppm) and n=9 (−82.8 ppm) at LH12ct‐SsirPW92/pcSseg‐3 level bracket the −74 ppm^[23]^ experimental shift value in solution and are clearly within the expected accuracy margin of the method.^[20]^ This corresponds to a remarkable solvent shift of more than 200 ppm.

The shifts at n=8, 9 are thought to realistically reflect the most probable situation in solution. This is borne out by additional computations for all clusters obtained in xTB meta‐dynamics simulations with 12 MeCN molecules, which we summarize in Figure S7 in Supporting Information. Energies at either the BP86‐D3/def2‐TZVPP/COSMO level used for the optimizations, or in LH12ct‐SsirPW92‐D3/pcSseg‐3/COSMO single‐point calculations to match the level used for the shift computations, clearly show that clusters with n=8, 9 provide the lowest energies, by about 10 kJ mol^−1^ compared to n=7 (the few n=6 clusters have even higher energies). The BP86‐D3 energies favor somewhat structures with n=8, giving shifts centered around ca. −65 ppm. The LH12ct‐SsirPW92‐D3 energies favor more structures with n=9, which lead to shifts closer to −80 ppm. I.e. the two coordination numbers give shifts slightly above or below the experimental value of −74 ppm and generally match experiment to within the accuracy of the method used in the shift computations. They are reasonably well represented by the single‐structure results shown in Figure 2, given that the shifts for a given n only show a small spread with different structures. This supports the approximation of using the “best” static cluster in this case, an approximation made also for the other ions, given that to carry out full MD simulations for all cases is beyond the scope of the present work. We note that during revision of this paper, Spicher et al.^[78]^ reported a new, automated workflow for the generation of microsolvated clusters, called “quantum cluster growth”. Such algorithms provide more freedom to include conformer averaging in a computationally affordable manner, and we will investigate its use in our ongoing work.

Shifts for DFT‐optimized clusters have been computed with increasing cluster size up to n=9 (Table S4, Figure 2). In Ref. [23] the solvent deshielding was linked to the larger shielding anisotropy in the monosolvated complexes used to model the effects. However, as we see in Table S5, the anisotropy only reflects the unsymmetrical coordination (see Figure 1 for some example cluster structures) and overall decreases with increasing coordination number, albeit in an uneven way due to the symmetry and structure of the given static solvate complex used. Yet, the isotropic shifts increase up to n=6, 7 before levelling off only slightly for n=8, 9 (Figure 2). Moreover, the computed shielding anisotropies for the larger clusters are much smaller (Table S4) than the overall solvent shifts (Table S4, Figure 2) and thus cannot be the major reason for the latter. Indeed, in isotropic solution the shielding anisotropy of a fluoride ion will vanish in the dynamical average. The mechanism by which solvation leads to low‐frequency shifts thus clearly must operate also in the absence of anisotropy, and we will analyze it in more detail below.

In aqueous solution, which we also studied for comparison, 3D‐RISM‐SCF suggests an average of six hydrogen bonds from water molecules to fluoride (see Figure S8), while previous ab initio MD simulations give results closer to n=5.^[79]^ Other microsolvated cluster models even suggest n=4 to be the predominant number of OH⋅⋅⋅F hydrogen bonds in the first solvation shell.^[80]^ Our computations on F^−^(H_2_O)_n_ cluster models embedded in COSMO‐RS surroundings show the Gibbs free energies of solvation to still drop after n=6, but in an irregular fashion (Figure S9, Table S6 in Supporting Information), even though closer inspection of the clusters suggest that indeed only four water molecules are coordinated to F^−^ in the larger clusters. This suggests that the contributions from the COSMO‐RS model are somewhat smaller than those contributed by adding more explicit solvent molecules to the clusters in the second solvation shell. However, the large scatter in the curve suggests that the static clusters in any case do not provide an as good description as they did for MeCN (see above). Free solvation energies approach the experimental range of around −460 kJ mol^−1^ (see above) but do not quite get there (Table S6, Figure S9). The “pure” COSMO‐RS value of −437.2 kJ mol^−1^ agrees well with the cluster‐based numbers but is also slightly too small in absolute value. We note in passing, that also in this case the free energies of the “monomer cycle” agree much less with experiment (Table S6) than those of the “cluster cycle” shown in Figure S9.

The ^19^F shifts increase sharply for small n and seem almost saturated at around n=6 (Figure S9, Table S7). At this n, the chosen cLH12ct‐SsirPW92/pcSseg‐3 level provides a shift of −150 ppm. Further deshielding by up to 10–15 ppm is seen for the larger n values. This brings us to around −130 ppm to −140 ppm, still somewhat below the experimental shift of −119 ppm of fluoride in aqueous solution.^[23]^ Overall, the solvent shift of around 140–150 ppm is in any case overall less pronounced than the more than 200 ppm we find for MeCN (see above). An explanation will be provided below.

Computations of F^−^ with only a COSMO or D‐COSMO‐RS solvent model fail completely to capture the solvent shifts, both in MeCN and in water. They give less than 2 ppm solvent shift for both solvents (−1.4 ppm in water for COSMO, −1.7 ppm for D‐COSMO‐RS). A 3D‐RISM‐SCF treatment of the solvents does not give any solvent effects on the ^19^F shifts. This is to be expected, since the spherical symmetry of the solute gives a spherical solvation potential, which in turn creates no polarization of the electron density of the fluoride ion compared to the gas phase.

That is, as previously noticed for the gas‐liquid ^17^O shift of water,^[32]^ electronic coupling of the solute and solvent orbitals in the magnetic field must play a decisive role for the solvent shifts in such strong‐interaction cases. We have analyzed these effects in more detail using the F^−^(MeCN)_8_ and F^−^(H_2_O)_6_ clusters (the latter cluster with S_6_ symmetry, taken from Ref. [23], is not a minimum but gives a good overall solvent shift and renders analyses relatively transparent). We first note that the solvent shifts arise purely from paramagnetic shielding contributions σ^p^ (Table S8). For further analyses, we have used a breakdown into orbital contributions using the implemented tools in the MAG code,^[71]^ at the quantitatively less accurate GIAO‐BP86/pcSseg‐2^[58]^ level (the more accurate functionals are not implemented in that code). For the F^−^(H_2_O)_6_ cluster, the deshielding compared to free fluoride ion is distributed over a substantial number of occupied canonical MOs (some MO plots are provided in Figure S10 in Supporting Information). All of them have some fluorine p‐orbital character mixed with various water oxygen‐based orbitals. This reflects the similar electronegativity of fluorine and oxygen, which places their valence orbitals in a similar energy range and thus leads to extensively delocalized canonical MOs for the cluster. While it confirms extensive solute‐solvent orbital mixing, further analyses in terms of occupied‐virtual MO couplings turn out to be complicated.

**Figure 3:**
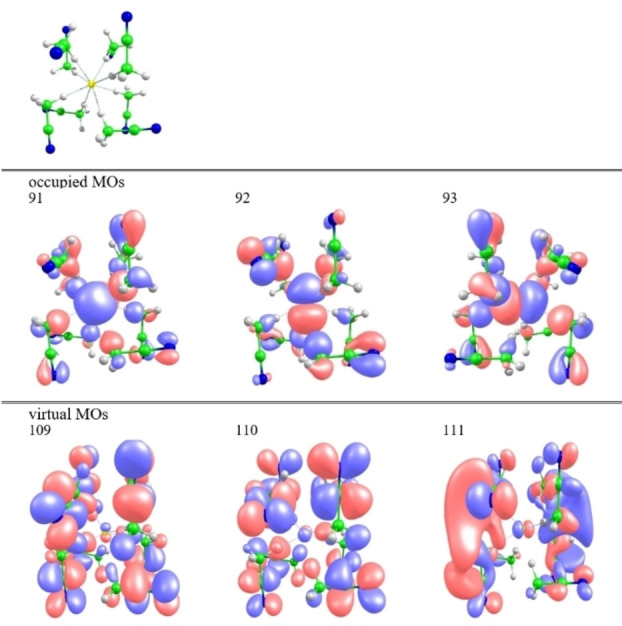
Structure of the optimized F^−^(MeCN)_8_ cluster used and isosurface plots (0.03 a.u.) of relevant occupied and virtual canonical molecular orbitals contributing to the ^19^F shielding tensor.

Analyses are more straightforward for the F^−^(MeCN)_8_ cluster, where the fluorine p‐orbital character concentrates more in the three highest, almost degenerate occupied canonical MOs, which indeed dominate the large deshielding compared to free fluoride (in particular the HOMO and HOMO‐1; see Figure 3 for MO plots). The dominant contributions to σ^p^ from occupied/virtual MO couplings arise from these three occupied MOs and from three virtual MOs. The latter do indeed have substantial σ*(C−H) character in the CH‐bonds coordinating to fluoride, albeit mixed with π*(C≡N) character of these coordinated MeCN molecules (Figure S10). Alternative analyses using Boys‐localized MOs^[81]^ within an IGLO^[72]^‐BP86‐based scheme confirm the dominance of occupied LMOs with fluorine p‐orbital character, but the couplings tend to be smeared over a larger number of (canonical) virtual MOs, rendering the analyses less transparent. Overall, our analyses clearly confirm couplings between fluorine lone pair orbitals and low‐lying solvent based virtuals for MeCN solvates. This holds also analogously for the interpretation of the ^19^F solvent shifts of the other species covered in this work (see below). We will not discuss extensive shielding analyses for these other species. We note here already that both the free solvation energies and the shift effects in MeCN are largest for fluoride compared to the solutes discussed further below.

### Bifluoride anion

Turning to the bifluoride ion FHF^−^, we note that its solvation in CH_2_Cl_2_ and CCl_4_ has been subject to recent AIMD simulations.^[22]^ While that work had its main focus on changes to the potential of the strong hydrogen bond within the solute, computed ^19^F shift results clearly also confirmed appreciable deshielding in both solvents even for symmetrical structures of the solute, on which we focus in the present work. Note that those authors found that the average coordination of CH bonds to the fluorine atoms tended to be close to two for each of the fluorine atoms,^[22]^ corresponding to n=4 in the present context. It was speculated that steric interactions between the coordinated solvent molecules determine average dihedral angles between their CH⋅⋅⋅F vectors and thereby limit the average coordination.

Here we focus on solvation in MeCN. 3D‐RISM‐SCF suggests an average number of CH⋅⋅⋅F interactions n=10 when integrating to the minimum after the second maximum (Figure S2b, the second maximum includes the sites coordinating to the other F atom, assuming 1 in 3 H sites to form a CH⋅⋅⋅F contact), that is, five hydrogen bonds per fluorine atom. Integrating only to the first minimum results in an average of 3 close interactions (again assuming 1 in 3 H sites to form a CH⋅⋅⋅F contact). The GFN2‐xTB preoptimizations and the subsequent DFT and DFT+COSMO optimizations with up to 12 solvent molecules agree with this notion, as maximally 10–11 short CH⋅⋅⋅F contacts below 2.3 Å are found, and there is a tendency of solvent molecules to bind in a second solvation shell when higher coordination numbers are explored (Table S1 in Supporting Information). The DLPNO‐SCS‐MP2‐based free energies behave similarly as for F^−^ but progress more slowly down to ca. −290 kJ mol^−1^ (Figure 2, Table S2), while DLPNO‐CCSD(T)‐F12 gives even about 20 kJ mol^−1^ more negative free energies. COSMO‐RS without explicit solvent molecules provides a somewhat less negative free solvation energy of −263 kJ mol^−1^, again (see above for fluoride) in agreement with the observation that the free energies of the microsolvated cluster models still grow more negative at n=14, even though this involves already solvent molecules in a second shell. That is, the “pure” COSMO‐RS treatment seems to underestimate slightly the overall binding. “Monomer‐cycle” free energies again exhibit an incorrect behavior (Table S3, Figures S4, S5)

The ^19^F NMR shifts tend to increase slowly up to n=10, 11 (Figure 2, Table S4), where they reach ca. −135 ppm (compared to experimental shifts of −145 ppm to −148 ppm^[82]^), a solvent shift of about +84 ppm compared to the gas‐phase anion (Table S4), less than half the solvent shift found for fluoride (see above). This may be compared to an average solvent ^19^F shift in CH_2_Cl_2_ of +79 ppm from the AIMD simulations in Ref. [22]. COSMO and D‐COSMO‐RS give a solvent shift of about 3 ppm, 3D‐RISM‐SCF of less than 2 ppm, both methods again failing completely to capture the orbital couplings and thus the explicit solvent effects on the shifts, as can be expected. Note that we have not varied the asymmetry of the F−H−F moiety, in contrast to the AIMD simulations in Ref. [22], which showed a coupling of an asymmetry in that nominally symmetrical hydrogen bond and an asymmetric solvation shell. We conclude thus that the bifluoride ion also exhibits large solvation shifts in MeCN, but less so than fluoride, consistent with the overall smaller binding/solvation energies.

### ClF_2_^−^ compared to ClF_4_^−^

For the ClF_2_^−^ anion, which originally stimulated our interest in these types of interactions of fluoride‐type fluorine atoms with the CH‐bonds of organic solvents,^[17]^ 3D‐RISM‐SCF gives a lower average number n=2 of close CH⋅⋅⋅F interactions (see also Figure S2, assuming 1 in 3 H sites to form a CH⋅⋅⋅F contact) than for the related FHF^−^ anion, where an identical analysis gives n=3 (see above). The second minimum in the RDF would accommodate 9 contacts. Overall, the RDF shows less pronounced structure than for FHF^−^. This trend is consistent with the cluster models, which provide only up to 6–7 short F⋅⋅⋅H distances below 2.3 Å, with additional solvent molecules moving further away from the fluorine sites (Table S1 in Supporting Information). The computed free energies for the microsolvated clusters nevertheless also go down further, even at n=12, beyond −260 kJ mol^−1^ (Figure 2, Table S2), where they have clearly not yet converged. “Pure” COSMO‐RS suggests a free solvation energy of −240.9 kJ mol^−1^, so the shape of the curve is again consistent with the microsolvation beyond the first solvent shell providing a slightly larger stabilization than the COSMO‐RS embedding. Overall the solvation energies are only slightly smaller than those discussed above for FHF^−^.

The solvent effects on the ^19^F NMR shifts still increase slowly up to n=11 (Figure 2, Table S3) where the calculations would suggest them to be about 73 ppm, also only slightly less than those for FHF^−^ (see above). However, possibly adding further solvent molecules to the second solvation shell beyond n=6, 7 leads to an unbalanced coordination sphere. It thus seems prudent to focus on the shifts obtained for n=6, 7. Then we arrive at shifts between −115 ppm and −121 ppm, which is reasonably close to the experimental shift of −125 ppm. This corresponds to a somewhat smaller and more realistic and still appreciable solvation shift of 47–53 ppm. COSMO and D‐COSMO‐RS give a solvent shift of −4.8 and +0.5 ppm, respectively, while 3D‐RISM‐SCF gives about +5 ppm. All of these methods are thus again unable to recover most of the actual solvent shifts. We note that our previous computations using COSMO(MeCN) in Ref. [17] for this and related EF_2_^−^ anions left deviations from the experimental shifts in solution of about 50–70 ppm using a similar local hybrid (LH12ct‐SsifPW92, still without current response). The present computations clearly show that this is due to the orbital couplings caused by specific CH⋅⋅⋅F hydrogen bonds not being covered by implicit or force‐field‐based solvent models.

In that same work, the ^19^F shifts of anions EF_4_^−^ in MeCN could be reproduced to within ca. 10 ppm using a COSMO model for the solvent.^[17]^ This suggests that the specific CH⋅⋅⋅F interactions are much less pronounced for the tetrafluoro species (as well as for the hexafluoro anions studied in the same paper), and it was argued that this reflects a less negative charge on fluorine (see below). 3D‐RISM‐SCF indeed suggests only an average of 1.5 close CH⋅⋅⋅F contacts for ClF_4_^−^ in MeCN overall for the four fluorine sites, and a much shallower RDF overall than for the other three anions (Figure S2). This is consistent with a much less structured solvation shell. While cluster models can be constructed for many more solvent molecules, they tend to feature only 6 short contacts below 2.3 Å (Table S1), about 8–10 below 2.5 Å. The free binding energies still go down at n=12 (DLPNO‐SCS‐MP2 ca. −251 kJ mol^−1^), but this also again involves second‐shell microsolvation (Figure 2, Table S2), and the “pure” COSMO‐RS value of −223.6 kJ mol^−1^ indicates again less binding. For both ClF_2_^−^ and ClF_4_^−^ the “monomer cycle” is again unsuitable (Table S3, Figures S3, S4).

The solvent effects on the ^19^F shifts in ClF_4_^−^ are much smaller than those for ClF_2_^−^, peaking at about 18 ppm at n=9, while at a more realistic n=6 the solvation shift is 10 ppm (Figure 2, Table S4), leading to a shift of 78 ppm compared to the experimental value of 67 ppm. This explains why even a COSMO‐based calculation without explicit inclusion of solvent molecules gave already reasonable agreement with experiment in Ref. [17]. In the present work, COSMO gives a solvent shift of −4.9 ppm, D‐COSMO‐RS −2.0 ppm, 3D‐RISM‐SCF (OPLS‐AA) ca. +11 ppm.

### NMR shifts of solvent nuclei

While the effects on the shifts of the coordinating MeCN molecules will be difficult or impossible to observe experimentally, due to the expected fast exchange on the NMR time scale and the likely dominance of the bulk solvent signals, we summarize the computed data nevertheless in Tables S9–S13 in Supporting Information. As one might expect, the effects due to the charge transfer from the anion to a given solvate molecule decrease with increasing cluster size, as the effects are somewhat “diluted” over more interactions. The ^1^H shifts of the coordinating hydrogen atoms are computed to have increased shifts compared to the noncoordinating ones by about 2–3 ppm for F^−^, by about 0.8 ppm for FHF^−^ and by about 0.4 ppm for ClF_2_^−^ (Tables S9, S10), while noncoordinating hydrogens show very small deviations from an (MeCN)_n_ cluster. The computed methyl and nitrile ^13^C shifts are within less than 1 ppm from a pure solvent cluster (CH_3_CN)_8_ (Tables S11, S12), the nitrile ^15^N shifts within less than 2.5 ppm (Table S13). Any ion‐induced shifts for these nuclei are thus even less likely to be observable experimentally.

### IR spectroscopic fingerprints for the interaction

**Figure 4:**
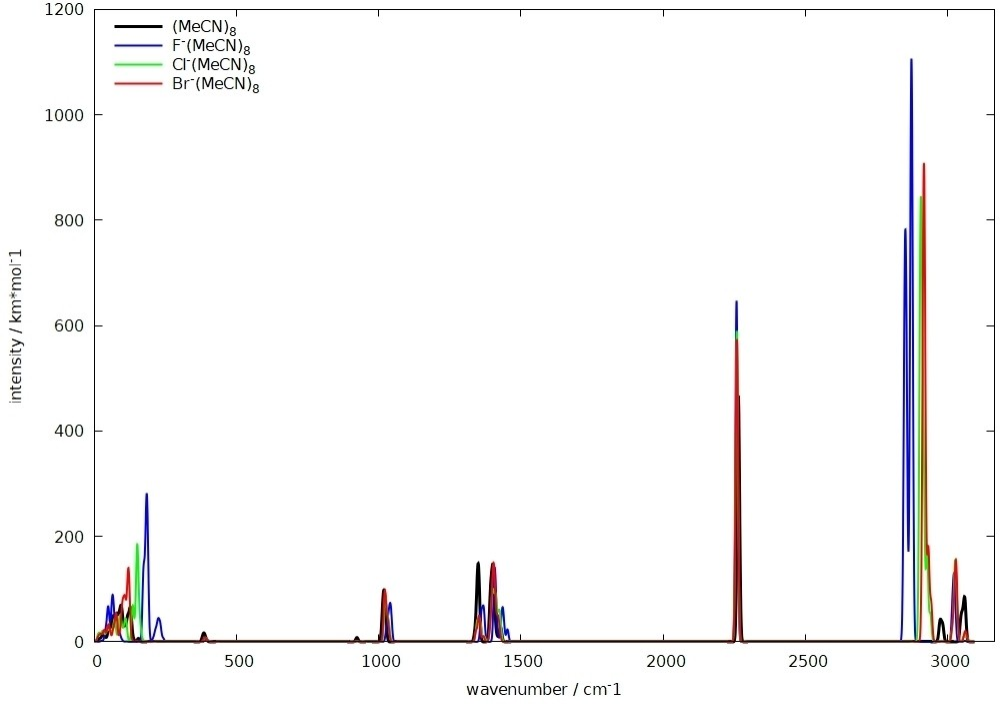
Computed harmonic IR Spectra of X^−^(MeCN)_8_ clusters (X=F, Cl, Br) in comparison with an (MeCN)_8_ cluster (BP86‐D3(BJ)/def2‐TZVPP/COSMO).

While weak CH⋅⋅⋅X hydrogen bonds tend to give blue‐shifted CH stretching frequencies in vibrational spectra,^[83]^ the larger charge transfer into the σ*(C−H) orbitals for stronger charge‐assisted interactions with anionic acceptors is expected to lead to strongly red‐shifted bands with enhanced intensity.[^5^, ^6^] This should certainly hold for simple halide ions in organic solvents, and the only question is, whether these shifted bands are sufficiently strong in comparison with the bulk spectra. As an exploratory computational examination, we have computed the harmonic vibrational spectra of the F^−^(MeCN)_8_ cluster in comparison with clusters of the heavier halides Cl^−^ and Br^−^ and a cluster (MeCN)_8_ without a halide ion as a rough approximation for the bulk liquid. The simulated spectra at BP86‐D3(BJ)/def2‐TZVPP/COSMO level are compared in Figure 4. It is clear that the halide ions generate red‐shifted, very intense C−H stretching bands that are not present in the (MeCN)_8_ cluster (nor in an isolated MeCN molecule). Both the red‐shift by several hundreds of cm^−1^ and the intensity increase by an order of magnitude are clearly most pronounced for fluoride. Such bands might well be observable even in the presence of the bulk vibrations. We note in passing that similar computational frequency and intensity shifts have also been found for molecular complexes of halide ions with fluoro‐ or chloroform, with fluoride again causing the largest effects.^[10d]^

### Comparison of charge transfer and polarization in F^−^(MeCN)_8_ and F^−^(H_2_O)_6_ clusters by NPA/NBO analyses

The fact that the ^19^F NMR solvent shifts of fluoride in MeCN are larger than in water prompted us to compare the amount of charge transfer in the respective cluster models by NBO analyses (BP86/pcSseg‐2//BP86‐D3(BJ)/def2‐TZVPP level, Table 1). Nuclear shieldings are a response property and thus do not necessarily follow the ground‐state charge on the NMR atom of interest.^[84]^ Still, such charge analyses can be useful. Interestingly, the negative NPA charge on fluorine is indeed smaller for the MeCN‐based cluster. However, the average charge transferred to one solvent ligand is slightly larger for the aqueous cluster (0.026 e) compared to the MeCN‐based cluster (0.023 e), and it is the larger number of solvent molecules that are able to coordinate for the organic solvent that overall depletes somewhat more charge from the fluoride than for the aqueous case (note that the actual n in water may be 4 or 5,[^79^, ^80^] so the disparity in the average coordination may even be slightly larger than for the chosen cluster sizes).

**Table 1:** Relevant NPA charges of atoms and fragments for the solvent complexes and the free solvent molecules (BP86/pcSseg‐2//BP86‐D3(BJ)/def2‐TZVPP)^[a]^

	MeCN	F^−^(MeCN)_8_		H_2_O	F^−^(H_2_O)_6_
Q(F)	–	−0.819	Q(F)	–	−0.843
Q(N)	−0.308	−0.368	Q(O)	−0.905	−0.975
Q(C1)	+0.263	+0.294	Q(H1)^[b]^	+0.452	+0.484
Q(C2)	−0.733	−0.785	Q(H2)	+0.452	+0.465
Q(H1)^[b]^	+0.259	+0.308			
Q(H2)	+0.259	+0.264			
Q(H3)	+0.260	+0.264			
Q(MeCN)	0.000	−0.023 (8x)	Q(H_2_O)	0.000	−0.026 (6x)

[a] Averaged over the eight and six solvent molecules, respectively. [b] Hydrogen atom coordinating to fluoride in the cluster.

For the latter, we see a redistribution of negative charge to the water oxygen atoms within the coordinated molecules, and the coordinating hydrogen ends up with an even more positive charge than for a free water molecule. This behavior is consistent with the behavior for a typical hydrogen bond, which enhances the ionicity of the bond within the hydrogen‐bond donor. That is, in addition to moderate charge transfer from the solute to the solvent molecules, charge becomes more polarized within the coordinating solvent, accumulating more negative charge on oxygen (which in turn will enhance further interactions with the next solvent shell, not covered by the cluster model). The same observations can be made for the MeCN case: in addition to the charge transfer from fluoride to the solvent molecules, the charge within the solvent molecules becomes more polarized, accumulating charge on the nitrogen and methyl carbon (C2) atom, while depleting charge from C1 and the hydrogen atoms, particularly on the coordinating ones (H1). This underscores the character of a strong CH⋅⋅⋅F hydrogen bond with an essentially conventional build. Perturbation‐theoretical analyses of the interactions between the strictly localized NBOs and the composition of the resulting natural localized MOs^[69]^ (NLMOs) confirm expectations that the charge transfer from fluoride to the solvent molecules involves donation into the σ*(H−O) and σ*(H−C) antibonding orbitals of the coordinating OH or CH units, respectively, consistent with the MO couplings responsible for the ^19^F solvation shifts (see above).

The interactions for the other anions are analogous albeit less pronounced, regarding both the charge‐transfer and the polarization within the MeCN molecules, consistent with the reduced negative fluorine NPA charges for the free gas‐phase anions of −0.756 (FHF^−^), −0.591 (ClF_2_^−^) and −0.529 (ClF_4_^−^).

## Conclusions

The element fluorine occupies a special place in the Periodic Table, not only as the most electronegative element but also due to its compact charge concentration within a small radial space. Once the accumulation of negative charge becomes very large, the resulting fluoride‐like units exhibit particularly strong CH⋅⋅⋅F hydrogen‐bonding interactions with the CH‐bonds of organic solvents, which are not captured by the usual continuum solvation models nor by force‐field‐based models. In this work we have largely concentrated on acetonitrile, but the effects are clearly observable for other CH‐bond containing solvents as well, CH_2_Cl_2_ or the haloforms being other examples.

Computational studies have already pointed out that anionic acceptors X^−^ can enhance CH⋅⋅⋅X^−^ interactions to the extent that they have to be considered strong, charge‐assisted hydrogen bonds (see literature pointed out in the Introduction). The focus of the present work has been on the extremely large and characteristic solvent effects on ^19^F NMR shifts generated by these types of hydrogen bonds. It had been known that ^19^F solvent shifts of fluoride in many organic solvents are larger than in aqueous solution. Our analyses show that this is largely due to the fact that a solvent like acetonitrile can form more CH⋅⋅⋅F hydrogen bonds to fluoride than possible in aqueous solution (8–9 rather than 4–6). In contrast to previous interpretations, it is this larger number of (slightly weaker) hydrogen‐bonding interactions that leads to a larger solvation shift, in spite of an overall somewhat smaller solvation free energy. This is why Christe had noted that the ^19^F shift of fluoride cannot be used as an indicator of its “nakedness”.

However, we do confirm that for a given solvent the ^19^F solvation shifts of different anionic fluorine species correlate with the magnitude of their solvation free energies, which are in turn determined by the strengths of the CH⋅⋅⋅F hydrogen bonds that correlate with the amount of negative charge on the given fluorine atom. That is, both the solvation ^19^F shifts and the solvation free energies in acetonitrile decrease along the series F^−^ ≫ FHF^−^ > ClF_2_^−^ > ClF_4_^−^ studied in the present work. The ^19^F solvation shifts reach astounding +227 ppm for F^−^ and clearly require a proper quantum‐chemical treatment of microsolvation to be reproduced accurately. In contrast, the effects of microsolvation are close to +10 ppm for ClF_4_^−^, where the negative charge is more delocalized and thus smaller on a given fluorine atom. In the latter case these specific solvation effects are still relatively close to the error margins of even rather advanced DFT approaches but might be needed accounting for in more accurate benchmark‐level treatments. And more delocalized fluoridic ions will exhibit even less pronounced effects of microsolvation. But already for ClF_2_^−^, the ^19^F solvation shifts of 50–60 ppm are far outside practically achievable DFT accuracies and thus clearly have to be dealt with.

None of the implicit or force‐field‐based solvent models studied here, COSMO, D‐COSMO‐RS or 3D‐RISM‐SCF, can reproduce these microsolvation effects on the ^19^F shifts, as these involve a coupling of solute and solvent orbitals in the magnetic‐field response. This clearly requires an accurate quantum‐mechanical treatment of the directly bound solvent molecules. In the present work, we have pre‐generated the static DFT cluster models used for the computation of both shifts and solvation free energies by cheap tight‐binding (xTB) meta‐dynamics methods available in Grimme's CREST program, followed by DFT optimizations. In the present case of relatively simple anionic species, this has still led to probably reasonable estimates of the microsolvation effects. Clearly, matters will become less tractable for routine computational treatment for more complicated systems that one may encounter in various chemical applications (also for other electronegative elements like oxygen or chlorine when they exhibit large negative charge). This puts more emphasis on improved and expedient methods for the generation of microsolvated clusters (as, for example, the recent QCG algorithm, see above), short of the costs of full AIMD simulations, to be used in mechanistic studies of chemical reactions where the “fluoridic” character may vary along a reaction coordinate. Thermochemistry and kinetics of such reactions are expected to be strongly affected by differential microsolvation contributions not only in protic but also in “aprotic” organic solvents. In contrast to the NMR shift case, in this case we have semi‐empirical methods like COSMO‐RS as reasonable albeit not perfect alternatives to obtain estimates of solvation free energies. Finally, microsolvated cluster models are also required to access computationally the fingerprints that such CH⋅⋅⋅X hydrogen bonds may cause in the vibrational frequencies and intensities of C−H vibrations of the coordinated solvent molecules.

## Supporting Information Summary

Tables with additional data on structures, thermochemistry and NMR chemical shifts, Figures on 3D‐RISM‐SCF radial distribution functions, cluster models, free energies and NMR shifts as functions of cluster size, relevant MOs for analyzing solvent NMR shifts.

## Conflict of interest

The authors declare no conflict of interest.

1

## Supporting information

As a service to our authors and readers, this journal provides supporting information supplied by the authors. Such materials are peer reviewed and may be re‐organized for online delivery, but are not copy‐edited or typeset. Technical support issues arising from supporting information (other than missing files) should be addressed to the authors.

Supporting Information

Supporting Information

## Data Availability

The data that support the findings of this study are available in the supplementary material of this article.
