# Supplementary material for: Unusually Large Effects of Charge‐assisted C−H⋅⋅⋅F Hydrogen Bonds to Anionic Fluorine in Organic Solvents: Computational Study of 19F NMR Shifts versus Thermochemistry
Source: ChemistryOpen. 2022 Aug 19;11(12):e202200146. doi: 10.1002/open.202200146 (PMC9716039; doi:10.1002/open.202200146)
Supplement: Supplementary file 1 — Supporting Information [file OPEN-11-e202200146-s001.pdf]

ChemistryOpen

Supporting Information

Unusually Large Effects of Charge-assisted C–H···F Hydrogen Bonds to Anionic Fluorine in Organic Solvents: Computational Study of ^{19}F NMR Shifts versus Thermochemistry

Martin Kaupp,* Caspar J. Schattenberg, Robert Müller, and Marc Reimann

Unusually large effects of charge-assisted C-H···F hydrogen bonds to anionic fluorine in organic solvents: computational study of ^{19}F NMR shifts vs. thermochemistry

-- Supporting Information --

M. Kaupp,* C. J. Schattenberg, R. Müller, Marc Reimann

Technische Universität Berlin, Institut für Chemie, Theoretische Chemie/Quantenchemie, Sekr. C7, Strasse des 17. Juni 135, 10623 Berlin (Germany).

**Email address: martin.kaupp@tu-berlin.de*

Contents

S1. Radial distribution plots from 3D-RISM-SCF in MeCN 2

S2. Structures of microsolvated clusters in MeCN.. 3

S3. Thermochemical results in MeCN 5

S4. NMR chemical shifts in MeCN 18

S5. Thermochemical data in water... 21

S6. Dependence of energies and shifts on cluster structure for F^- in MeCN 23

S7. Further results for F^- in aqueous solution.. 24

S8. Analyses of solvent effects on NMR chemical shifts 27

S9. NMR shifts of coordinated and uncoordinated MeCN solvent molecules 30

S1. Radial distribution plots from 3D-RISM-SCF in MeCN

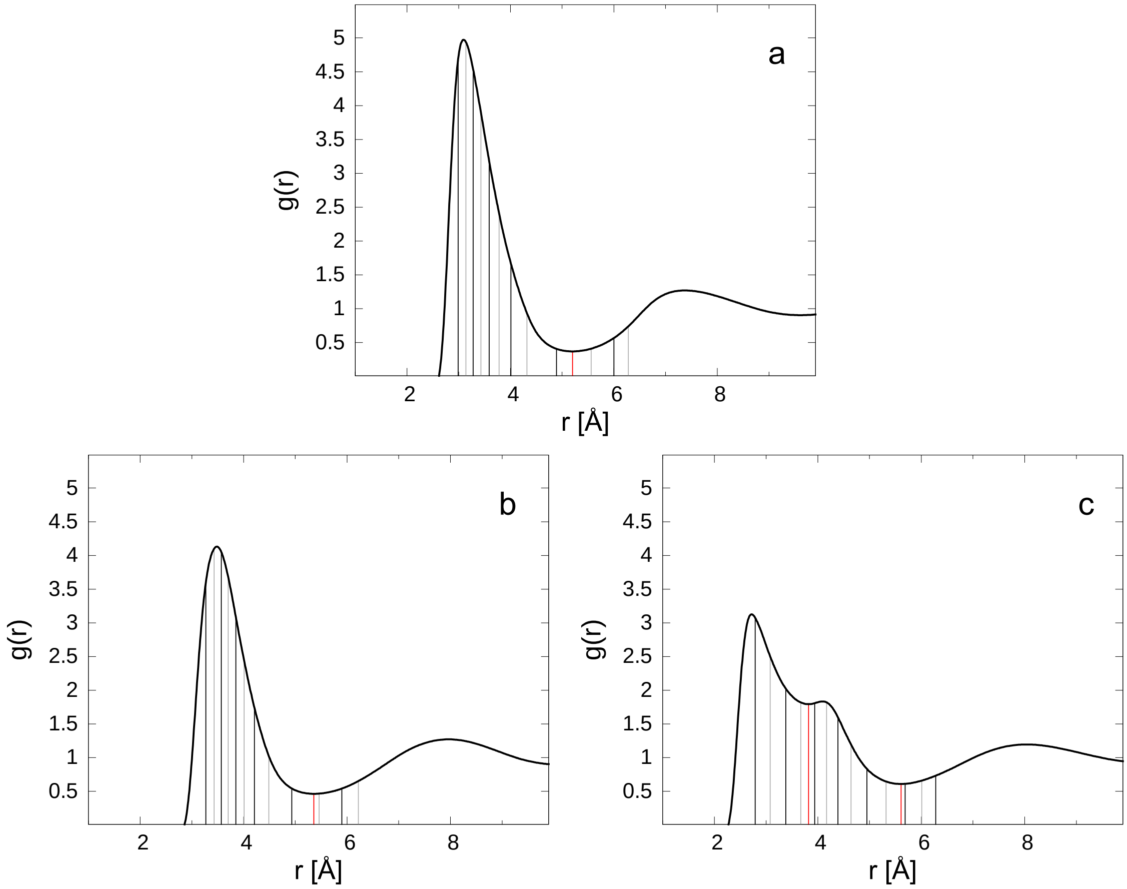

Figure S1. Spherically averaged pair distribution functions from 3D-RISM-SCF calculations of the fluoride ion in MeCN. We show (a) the F···Me(UA), (b) the F···C(H_3) and (c) the F···H (c) distribution functions. The vertical lines mark the positions, up to which g(r) integrates to an integer number of MeCN molecules, the red lines mark minima.

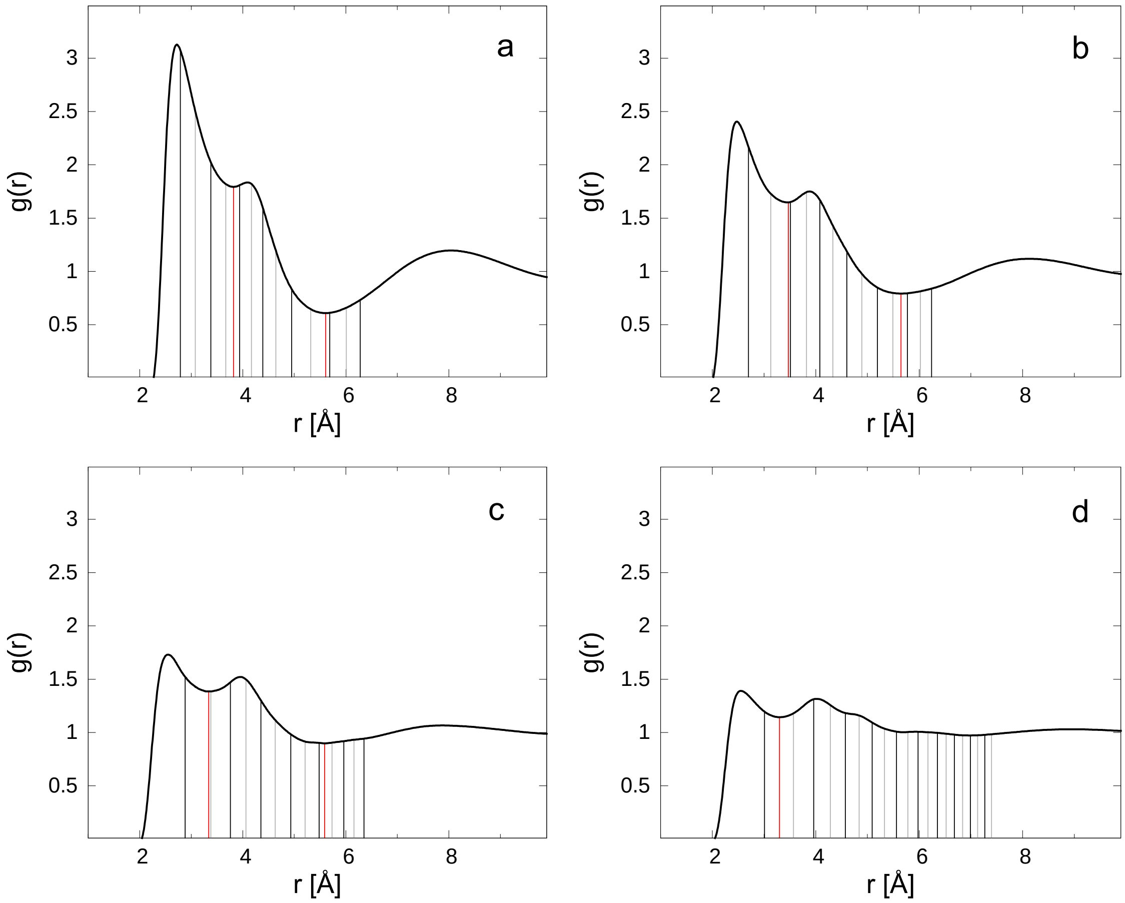

Figure S2. Spherically averaged pair distribution functions from 3D-RISM-SCF calculations of anions in MeCN. We show the F···H distribution functions of (a) F^- , (b) FHF^-, (c) ClF_2^-, (d) ClF_4^-. The vertical lines mark the positions, up to which g(r) integrates to an integer number of MeCN molecules, the red lines mark minima.

S2. Structures of microsolvated clusters in MeCN

Table S1. Optimized CH…F bond lengths in pm in optimized $X^{-}(MeCN)_n$ ($X = F^{-}$, FHF^{-}, ClF_2^{-}, ClF_4^{-}) clusters at GFN2-xTB, MARIJ-BP86-D3(BJ)/def2-TZVPP levels (DFT+C uses COSMO, ε = 35.688) levels.

Anion	n	XTB	DFT	DFT+C	Anion	n	XTB	DFT	DFT+C
F^{-}	8	198.9	197.5	198.2	FHF^{-}	10	210.5	206.7	205.4
		198.9	197.5	198.2			219.3	208.3	206.6
		198.9	197.5	199.7			220.0	210.0	206.6
		198.9	197.5	199.7			220.7	213.1	207.4
		214.0	209.0	202.6			221.2	213.4	207.5
		214.0	209.0	202.6			221.5	213.7	208.6
		214.0	209.0	203.2			223.0	215.8	209.5
		214.0	209.0	203.2			223.2	216.4	214.8
							227.2	218.3	(233.9)
							238.4	218.7	(272.2)
	9	201.2	204.9	204.0		11	217.5	208.2	205.9
		201.2	204.9	204.2			220.0	209.5	208.0
		201.8	205.0	204.2			223.3	210.9	210.7
		201.8	211.8	206.6			223.4	211.5	211.5
		206.0	211.9	206.7			224.8	211.9	212.0
		206.0	212.0	206.7			226.1	212.5	212.8
		210.1	214.4	216.1			226.4	213.3	213.2
		213.1	214.5	216.4			227.1	213.8	215.7
		(505.9)	214.5	216.5			228.6	220.9	217.8
							228.6	221.5	218.1
							(231.7)	(513.3)	(484.4)
	10	193.9	192.9	196.0		12	218.7	212.0	210.5
		194.6	199.8	198.3			220.2	214.1	211.4
		202.2	202.5	198.9			222.3	214.8	212.9
		202.6	203.5	202.2			222.4	215.2	215.0
		211.2	203.8	202.5			223.3	217.0	215.8
		212.8	204.0	203.0			225.9	217.0	216.9
		212.9	205.9	203.9			227.7	218.9	218.5
		215.3	206.9	204.2			227.7	220.5	219.0
		(540.6)	(506.2)	(462.5)			228.2	222.3	221.8
		(574.1)	(565.5)	(585.4)			(230.5)	225.2	227.0
							(234.6)	228.1	(235.9)
							(479.0)	(472.3)	(443.6)
ClF_2^{-}	10	218.0	208.0	209.1	ClF_4^{-}	10	222.3	217.4	215.8
		218.7	208.5	210.1			225.8	222.5	221.6
		219.2	209.2	212.1			226.1	222.7	222.4
		221.8	213.1	213.2			(233.4)	225.9	224.7
		(231.4)	214.1	215.6			(235.9)	228.2	228.8
		(235.4)	217.9	217.5			(238.2)	(237.7)	229.8
		(237.9)	220.8	220.3			(240.9)	(239.7)	(238.1)
		(243.0)	(254.6)	259.6			(244.7)	(243.0)	(242.6)
		(246.3)	(267.6)	265.4			(264.1)	(290.5)	(257.8)
		(247.7)	(299.1)	(315.6)			(284.4)	(294.3)	(280.2)
	11	214.5	209.1	206.6		11	226.7	211.9	215.0
		217.9	209.8	208.8			227.8	224.4	224.8
		218.8	212.5	212.8			(232.1)	226.0	226.2
		220.1	214.4	213.2			(232.2)	(231.1)	229.8
		223.1	214.4	214.6			(234.9)	(244.3)	229.8
		226.4	215.8	215.4			(236.8)	(245.5)	(233.1)
		(239.6)	223.1	220.5			(242.0)	(246.3)	(236.3)
		(259.7)	223.8	(247.5)			(248.6)	(247.6)	(241.5)
		(336.0)	(242.2)	(260.4)			(251.7)	(249.1)	(242.9)
		(342.2)	(283.9)	(277.1)			(257.1)	(251.1)	(246.3)
		(360.1)	(297.9)	(289.6)			(258.3)	(259.3)	(261.5)
	12	218.2	208.9	209.6		12	223.0	217.0	217.7
		219.2	208.9	209.7			223.0	217.1	217.8
		221.2	212.3	209.8			(233.6)	221.2	222.9
		222.6	212.4	209.9			(233.6)	221.2	223.0
		224.4	212.6	214.3			(235.8)	222.6	227.9
		225.5	212.7	214.6			(235.8)	222.7	227.9
		(230.4)	(233.4)	(236.1)			(239.4)	229.9	(231.5)
		(234.6)	(234.6)	(238.8)			(239.4)	(230.6)	(231.5)
		(274.2)	(291.0)	(284.6)			(248.9)	(233.9)	(232.0)
		(297.1)	(291.6)	(284.8)			(248.9)	(233.9)	(232.0)
		(362.9)	(292.7)	(290.3)			(252.0)	(247.4)	(249.2)
		(368.6)	(292.8)	(291.0)			(252.0)	(248.0)	(249.2)

S3. Thermochemical results in MeCN

Table S2. Cluster-cycle energetics: computed gas-phase binding energies ($\Delta E_{bind,gp}$), binding enthalpies ($\Delta H_{bind,gp}$), Gibbs free binding energies ($\Delta G_{bind,gp}$), and final solvation Gibbs free energies $\Delta G_{solv}(X^-)$ of anions X^- in acetonitrile ($X^- = ClF_2^-$, ClF_4^-, FHF^-, F^-) using explicit $(MeCN)_n$ clusters at DFT (BP86-D3(BJ)/def2-TZVPP, ωB97M-V/def2-TZVPP), DLPNO-SCS-MP2/aug-cc-pVTZ, and DLPNO-CCSD(T)-F12/cc-pVTZ-F12 level.[a] Thermal and entropic contributions have been evaluated at standard state conditions (298.15 K, 0.1 MPa).[b] $\Delta\Delta G_{solv}$ is the bulk solvent free energy change obtained from COSMO-RS computations,[c] SScorr is the standard state correction,[d] and $-T\Delta S$ is the entropic contribution term.[b] All energies are given in kJ mol^{-1}.

					BP86-D3(BJ)				ωB97M-V			
**Anion**	***n***[e]	**$\Delta\Delta G_{solv}$**	**SScorr**	**-TΔS**	**$\Delta E_{bind,gp}$**	**$\Delta H_{bind,gp}$**	**$\Delta G_{bind,gp}$**	**$\Delta G_{solv}(X^-)$**	**$\Delta E_{bind,gp}$**	**$\Delta H_{bind,gp}$**	**$\Delta G_{bind,gp}$**	**$\Delta G_{solv}(X^-)$**
**FHF^-**	**1**	-228.3	-7.9	30.7	-86.9	-84.7	-54.0	-290.2	-84.8	-82.6	-51.9	-288.2
	**2**	-191.7	-7.9	29.1	-127.9	-126.0	-97.0	-296.6	-127.9	-126.0	-96.9	-296.6
	**3**	-190.1	-7.9	38.4	-157.7	-155.3	-116.9	-314.8	-162.1	-159.7	-121.3	-319.3
	**4**	-160.0	-7.9	22.3	-165.8	-161.8	-139.5	-307.4	-168.2	-164.2	-141.9	-309.8
	**5**	-163.4	-7.9	39.4	-186.4	-181.4	-142.0	-313.4	-183.0	-178.0	-138.6	-309.9
	**6**	-148.1	-7.9	38.8	-193.2	-187.3	-148.5	-304.5	-190.2	-184.2	-145.4	-301.4
	**7**	-136.8	-7.9	31.4	-206.9	-201.5	-170.1	-314.8	-206.4	-201.0	-169.6	-314.3
	**8**	-136.2	-7.9	34.8	-212.8	-210.1	-175.2	-319.4	-213.7	-211.0	-176.2	-320.3
	**9**	-133.0	-7.9	37.7	-219.7	-216.5	-178.7	-319.6	-220.9	-217.7	-180.0	-320.9
	**10**	-120.8	-7.9	33.9	-217.5	-214.0	-180.2	-308.9	-219.9	-216.5	-182.6	-311.3
	**11**	-132.9	-7.9	47.3	-228.5	-225.0	-177.7	-318.5	-232.9	-229.4	-182.0	-322.9
	**12**	-123.8	-7.9	43.2	-230.8	-227.1	-183.9	-315.6	-236.1	-232.5	-189.2	-321.0
	**13**	-133.9	-7.9	47.4	-226.7	-223.0	-175.6	-317.4	-236.1	-232.4	-185.0	-326.8
	**14**	-131.0	-7.9	29.9	-221.9	-218.8	-188.9	-327.8	-231.9	-228.8	-198.9	-337.8
	**0**							-263.7				-263.7
**F^-**	**1**	-246.3	-7.9	30.0	-165.9	-176.7	-146.7	-400.9	-144.2	-155.0	-125.0	-379.2
	**2**	-198.2	-7.9	23.7	-220.4	-227.9	-204.3	-410.4	-198.0	-205.5	-181.9	-388.0
	**3**	-186.3	-7.9	40.7	-265.6	-270.8	-230.1	-424.4	-247.6	-252.8	-212.2	-406.4
	**4**	-150.7	-7.9	18.5	-285.3	-289.9	-271.3	-429.9	-266.8	-271.4	-252.9	-411.5
	**5**	-145.8	-7.9	30.3	-313.4	-316.6	-286.3	-440.1	-296.7	-299.9	-269.6	-423.4
	**6**	-150.1	-7.9	40.6	-327.7	-329.4	-288.8	-446.9	-313.5	-315.3	-274.7	-432.7
	**7**	-135.9	-7.9	30.0	-339.8	-340.7	-310.7	-454.4	-325.7	-326.6	-296.6	-440.4
	**8**	-128.7	-7.9	32.7	-347.4	-347.1	-314.4	-451.0	-335.7	-335.3	-302.7	-439.3
	**9**	-135.3	-7.9	48.8	-354.0	-352.4	-303.6	-446.8	-344.5	-342.9	-294.1	-437.3
	**10**	-128.0	-7.9	36.1	-348.8	-348.7	-312.6	-448.5	-335.1	-334.9	-298.9	-434.7
	**0**							-375.2				-375.2

Table S2 cont….

					DLPNO-SCS-MP2				DLPNO-CCSD(T)-F12			
Anion	n^e	$\Delta\Delta G_{solv}$	SScorr	-TΔS	$\Delta E_{bind,gp}$	$\Delta H_{bind,gp}$	$\Delta G_{bind,gp}$	$\Delta G_{solv}(X^-)$	$\Delta E_{bind,gp}$	$\Delta H_{bind,gp}$	$\Delta G_{bind,gp}$	$\Delta G_{solv}(X^-)$
FHF^-	1	-228.3	-7.9	30.7	-61.9	-59.6	-28.9	-265.2	-67.9	-65.6	-34.9	-271.2
	2	-191.7	-7.9	29.1	-93.5	-91.6	-62.6	-262.2	-101.9	-100.0	-70.9	-270.6
	3	-190.1	-7.9	38.4	-122.4	-120.0	-81.5	-279.5	-132.1	-129.7	-91.3	-289.3
	4	-160.0	-7.9	22.3	-126.2	-122.2	-99.9	-267.9	-137.3	-133.3	-111.0	-278.9
	5	-163.4	-7.9	39.4	-143.9	-138.9	-99.5	-270.9	-154.6	-149.6	-110.2	-281.6
	6	-148.1	-7.9	38.8	-150.4	-144.4	-105.6	-261.6	-161.3	-155.4	-116.6	-272.6
	7	-136.8	-7.9	31.4	-162.6	-157.3	-125.8	-270.5	-177.5	-172.2	-140.7	-285.4
	8	-136.2	-7.9	34.8	-167.9	-165.2	-130.3	-274.5	-183.5	-180.8	-145.9	-290.1
	9	-133.0	-7.9	37.7	-176.1	-172.9	-135.1	-276.0	-188.6	-185.4	-147.6	-288.5
	10	-120.8	-7.9	33.9	-173.0	-169.5	-135.7	-264.4	-190.5	-187.0	-153.1	-281.8
	11	-132.9	-7.9	47.3	-186.1	-182.6	-135.3	-276.1				
	12	-123.8	-7.9	43.2	-189.0	-185.3	-142.1	-273.8				
	13	-133.9	-7.9	47.4	-189.4	-185.7	-138.3	-280.2				
	14	-131.0	-7.9	29.9	-185.6	-182.4	-152.6	-291.4				
	0							-263.7				-263.7
F^-	1	-246.3	-7.9	30.0	-82.4	-93.2	-63.2	-317.4	-93.3	-104.0	-74.0	-328.3
	2	-198.2	-7.9	23.7	-134.6	-142.2	-118.5	-324.6	-148.0	-155.5	-131.8	-338.0
	3	-186.3	-7.9	40.7	-179.1	-184.3	-143.7	-337.9	-194.0	-199.2	-158.5	-352.8
	4	-150.7	-7.9	18.5	-194.7	-199.3	-180.8	-339.4	-211.7	-216.3	-197.8	-356.4
	5	-145.8	-7.9	30.3	-222.9	-226.0	-195.7	-349.5	-242.0	-245.1	-214.9	-368.6
	6	-150.1	-7.9	40.6	-235.3	-237.0	-196.4	-354.5	-254.0	-255.8	-215.2	-373.2
	7	-135.9	-7.9	30.0	-249.5	-250.4	-220.4	-364.2	-270.7	-271.6	-241.6	-385.4
	8	-128.7	-7.9	32.7	-259.6	-259.2	-226.5	-363.2	-280.2	-279.9	-247.2	-383.8
	9	-135.3	-7.9	48.8	-270.2	-268.6	-219.8	-363.0	-284.9	-283.3	-234.6	-377.7
	10	-128.0	-7.9	36.1	-258.3	-258.1	-222.1	-357.9	-279.6	-279.5	-243.4	-379.3
	0							-375.2				-375.2

Table S2 cont….

Anion	n^e	$\Delta\Delta G_{solv}$	SScorr	-T∆S	BP86-D3(BJ) $\Delta E_{bind,gp}$	BP86-D3(BJ) $\Delta H_{bind,gp}$	BP86-D3(BJ) $\Delta G_{bind,gp}$	BP86-D3(BJ) $\Delta G_{solv}(X^-)$	ωB97M-V $\Delta E_{bind,gp}$	ωB97M-V $\Delta H_{bind,gp}$	ωB97M-V $\Delta G_{bind,gp}$	ωB97M-V $\Delta G_{solv}(X^-)$
ClF_2^-	1	-215.3	-7.9	30.3	-65.5	-62.8	-32.5	-255.7	-65.6	-62.9	-32.6	-255.8
	2	-182.5	-7.9	24.7	-93.7	-91.4	-66.7	-257.1	-95.8	-93.5	-68.8	-259.2
	3	-192.9	-7.9	48.2	-117.5	-114.6	-66.3	-267.1	-120.3	-117.3	-69.1	-269.9
	4	-165.4	-7.9	33.7	-125.9	-123.0	-89.3	-262.6	-125.6	-122.7	-89.0	-262.3
	5	-162.9	-7.9	39.9	-143.3	-140.1	-100.2	-271.1	-145.4	-142.2	-102.3	-273.1
	6	-156.7	-7.9	42.6	-147.4	-143.9	-101.3	-265.9	-149.9	-146.3	-103.7	-268.4
	7	-138.4	-7.9	34.0	-159.5	-156.1	-122.1	-268.4	-160.1	-156.7	-122.7	-269.0
	8	-135.4	-7.9	34.2	-166.4	-162.7	-128.5	-271.8	-172.7	-169.0	-134.8	-278.1
	9	-141.6	-7.9	46.0	-173.0	-169.2	-123.3	-272.8	-177.5	-173.7	-127.8	-277.3
	10	-134.4	-7.9	47.7	-172.4	-171.1	-123.4	-265.8	-180.4	-179.1	-131.5	-273.8
	11	-129.5	-7.9	39.6	-183.1	-179.3	-139.6	-277.1	-192.4	-188.6	-148.9	-286.4
	12	-137.4	-7.9	42.7	-190.0	-186.3	-143.6	-288.9	-199.1	-195.4	-152.7	-298.0
	13	-132.1	-7.9	46.0	-188.4	-184.5	-138.5	-278.5	-199.8	-195.9	-150.0	-290.0
	14	-143.9	-7.9	36.8	-179.6	-175.8	-139.0	-290.9	-199.1	-195.3	-158.5	-310.4
	0							-240.9				-240.9
ClF_4^-	1	-201.7	-7.9	34.4	-57.0	-52.3	-17.9	-227.5	-59.9	-55.3	-20.9	-230.5
	2	-180.8	-7.9	32.1	-80.1	-75.6	-43.5	-232.3	-86.1	-81.6	-49.5	-238.3
	3	-179.8	-7.9	44.9	-97.5	-92.7	-47.8	-235.5	-106.6	-101.7	-56.9	-244.6
	4	-163.0	-7.9	38.2	-101.2	-99.0	-60.8	-231.7	-109.3	-107.1	-68.9	-239.8
	5	-157.1	-7.9	38.7	-114.6	-109.8	-71.1	-236.1	-124.7	-119.8	-81.1	-246.1
	6	-151.2	-7.9	39.1	-116.7	-111.9	-72.8	-231.9	-129.5	-124.7	-85.6	-244.7
	7	-142.4	-7.9	31.2	-127.8	-123.0	-91.8	-242.1	-140.2	-135.4	-104.2	-254.5
	8	-137.6	-7.9	32.9	-130.8	-125.7	-92.8	-238.3	-144.9	-139.7	-106.8	-252.3
	9	-140.0	-7.9	38.9	-133.8	-128.6	-89.7	-237.6	-148.2	-143.0	-104.1	-252.0
	10	-133.9	-7.9	33.3	-133.2	-128.2	-94.9	-236.7	-151.7	-146.6	-113.3	-255.1
	11	-135.9	-7.9	32.7	-143.9	-139.1	-106.4	-250.3	-170.4	-165.6	-133.0	-276.8
	12	-135.3	-7.9	39.8	-152.3	-146.7	-106.9	-250.2	-177.4	-171.8	-132.0	-275.2
	13	-133.0	-7.9	36.4	-148.3	-143.2	-106.8	-247.7	-174.0	-168.8	-132.4	-273.3
	14	-141.9	-7.9	29.2	-142.8	-137.7	-108.5	-258.3	-171.8	-166.7	-137.5	-287.3
	0							-223.6				-223.6

Table S2 cont….

Anion	n^e	ΔΔG_{solv}	SScorr	-TΔS	DLPNO-SCS-MP2				DLPNO-CCSD(T)-F12			
					Δ$E_{bind,gp}$	Δ$H_{bind,gp}$	Δ$G_{bind,gp}$	Δ$G_{solv}(X^-)$	Δ$E_{bind,gp}$	Δ$H_{bind,gp}$	Δ$G_{bind,gp}$	Δ$G_{solv}(X^-)$
ClF_2^-	1	-215.3	-7.9	30.3	-54.2	-51.4	-21.1	-244.4	-57.6	-54.9	-24.6	-247.9
	2	-182.5	-7.9	24.7	-78.8	-76.5	-51.7	-242.1	-84.5	-82.2	-57.5	-247.8
	3	-192.9	-7.9	48.2	-100.7	-97.8	-49.5	-250.3	-106.8	-103.9	-55.7	-256.5
	4	-165.4	-7.9	33.7	-104.9	-102.0	-68.2	-241.6	-111.1	-108.3	-74.5	-247.9
	5	-162.9	-7.9	39.9	-123.0	-119.7	-79.8	-250.7	-130.8	-127.6	-87.7	-258.6
	6	-156.7	-7.9	42.6	-126.8	-123.3	-80.6	-245.3	-134.9	-131.4	-88.7	-253.4
	7	-138.4	-7.9	34.0	-133.3	-129.9	-95.8	-242.1	-146.8	-143.4	-109.3	-255.7
	8	-135.4	-7.9	34.2	-146.0	-142.3	-108.1	-251.3	-157.7	-154.0	-119.8	-263.1
	9	-141.6	-7.9	46.0	-152.2	-148.4	-102.4	-252.0	-159.4	-155.6	-109.7	-259.2
	10	-134.4	-7.9	47.7	-152.2	-150.9	-103.3	-245.6	-163.9	-162.7	-115.0	-257.3
	11	-129.5	-7.9	39.6	-165.2	-161.4	-121.7	-259.2				
	12	-137.4	-7.9	42.7	-171.6	-167.9	-125.2	-270.5				
	13	-132.1	-7.9	46.0	-171.3	-167.4	-121.5	-261.4				
	14	-143.9	-7.9	36.8	-170.0	-166.2	-129.4	-281.3				
	0							-240.9				-240.9
ClF_4^-	1	-201.7	-7.9	34.4	-50.4	-45.7	-11.3	-220.9	-52.8	-48.1	-13.7	-223.4
	2	-180.8	-7.9	32.1	-70.7	-66.3	-34.2	-222.9	-74.5	-70.1	-38.0	-226.7
	3	-179.8	-7.9	44.9	-89.8	-84.9	-40.0	-227.8	-92.7	-87.8	-43.0	-230.7
	4	-163.0	-7.9	38.2	-92.6	-90.4	-52.1	-223.1	-92.8	-90.5	-52.3	-223.3
	5	-157.1	-7.9	38.7	-104.4	-99.6	-60.9	-225.9	-111.0	-106.2	-67.5	-232.5
	6	-151.2	-7.9	39.1	-108.2	-103.4	-64.4	-223.4	-113.0	-108.2	-69.1	-228.2
	7	-142.4	-7.9	31.2	-118.3	-113.5	-82.3	-232.6	-124.8	-119.9	-88.7	-239.0
	8	-137.6	-7.9	32.9	-121.5	-116.3	-83.4	-228.9	-131.1	-125.9	-93.0	-238.5
	9	-140.0	-7.9	38.9	-126.6	-121.4	-82.5	-230.4	-130.0	-124.7	-85.8	-233.7
	10	-133.9	-7.9	33.3	-129.6	-124.6	-91.3	-233.1				
	11	-135.9	-7.9	32.7	-144.9	-140.1	-107.4	-251.3				
	12	-135.3	-7.9	39.8	-153.5	-147.9	-108.1	-251.3				
	13	-133.0	-7.9	36.4	-150.5	-145.3	-108.9	-249.8				
	14	-141.9	-7.9	29.2	-149.5	-144.4	-115.2	-265.0				
	0							-223.6				-223.6

Table S2 cont….

[a] Total electronic energies at the indicated levels were obtained by corresponding single-point calculations at MARIJ-BP86-D3(BJ)/def2-TZVPP gas-phase optimized structures.

[b] Thermal and entropic contributions at standard state conditions (298.15 K, 0.1 MPa) were evaluated within the ideal gas and rigid rotor – harmonic oscillator approximation based on harmonic vibrational frequency calculations at MARIJ-BP86-D3(BJ)/def2-TZVPP level.

[c] $\Delta\Delta G_{solv} = \Delta G_{solv}(X^{-}(MeCN)_{n}) - \Delta G_{solv}((MeCN)_{n})$; COSMO-RS solvation free energies were calculated by single-point calculations at MARIJ-BP86/def2-TZVPD[/COSMO(MeCN)] level at MARIJ-BP86-D3(BJ)/def2-TZVPP[/COSMO(MeCN)] optimized structures.

[d] SScorr = $-RT\ln(V_m)$; V_m = 24.46 mol L^{-1}.

[e] Index 0 indicates the COSMO-RS solvation free energy of X^{-} calculated without the inclusion of explicit solvent molecules, see the Computational Details.

Table S3. Monomer-cycle energetics: computed gas phase binding energies ($\Delta E_{bind,gp}$), binding enthalpies ($\Delta H_{bind,gp}$), free binding energies ($\Delta G_{bind,gp}$), and final solvation free energies $\Delta G_{solv}(X^-)$ of anions X^- in acetonitrile (X^- = ClF_2^-, ClF_4^-, FHF^-, F^-), using *n* distinct MeCN molecules for microsolvation, at DFT (BP86-D3(BJ)/def2-TZVPP, ωB97M-V/def2-TZVPP), DLPNO-SCS-MP2/aug-cc-pVTZ, and DLPNO-CCSD(T)-F12/cc-pVTZ-F12 level.[a] Thermal and entropic contributions have been evaluated at standard state conditions (298.15 K, 0.1 MPa).[b] $\Delta\Delta G_{solv}$ is the bulk solvent free energy change obtained from COSMO-RS computations,[c] SScorr is the standard state correction,[d] and –T ΔS is the entropic contribution term.[b] All energies are given in kJ mol^{-1}.

					BP86-D3(BJ)				ωB97M-V			
**Anion**	***n***[e]	**$\Delta\Delta G_{solv}$**	**SScorr**	**-TΔS**	**$\Delta E_{bind,gp}$**	**$\Delta H_{bind,gp}$**	**$\Delta G_{bind,gp}$**	**$\Delta G_{solv}(X^-)$**	**$\Delta E_{bind,gp}$**	**$\Delta H_{bind,gp}$**	**$\Delta G_{bind,gp}$**	**$\Delta G_{solv}(X^-)$**
**FHF^-**	**1**	-228.3	-7.9	30.7	-86.9	-84.7	-54.0	-290.2	-84.8	-82.6	-51.9	-288.2
	**2**	-197.4	-15.9	62.0	-153.7	-146.7	-84.7	-297.9	-154.6	-147.6	-85.5	-298.8
	**3**	-185.8	-23.8	101.0	-208.1	-195.5	-94.5	-304.0	-211.4	-198.7	-97.8	-307.3
	**4**	-168.4	-31.7	137.1	-256.8	-237.2	-100.2	-300.2	-261.9	-242.2	-105.2	-305.3
	**5**	-169.5	-39.6	186.3	-302.0	-276.1	-89.9	-299.0	-301.2	-275.3	-89.1	-298.2
	**6**	-162.5	-47.6	227.2	-346.5	-314.4	-87.3	-297.4	-348.5	-316.5	-89.3	-299.4
	**7**	-155.9	-55.5	268.4	-390.1	-353.2	-84.8	-296.2	-394.7	-357.8	-89.5	-300.8
	**8**	-156.2	-63.4	313.2	-431.4	-392.0	-78.9	-298.5	-437.9	-398.6	-85.4	-305.0
	**9**	-148.5	-71.3	347.7	-474.3	-429.4	-81.7	-301.6	-482.1	-437.1	-89.4	-309.3
	**10**	-141.6	-79.3	394.2	-511.9	-461.1	-67.0	-287.9	-522.9	-472.1	-78.0	-298.8
	**11**	-149.2	-87.2	441.6	-552.8	-496.9	-55.3	-291.6	-565.3	-509.3	-67.8	-304.1
	**12**	-134.5	-95.1	482.6	-591.3	-529.6	-47.0	-276.6	-602.5	-540.8	-58.3	-287.9
	**13**	-150.2	-103.0	531.7	-626.2	-559.4	-27.7	-280.9	-643.6	-576.8	-45.1	-298.3
	**14**	-137.2	-111.0	564.5	-662.0	-590.0	-25.6	-273.7	-676.1	-604.2	-39.7	-287.9
	**0**							-263.7				-263.7
**F^-**	**1**	-246.3	-7.9	30.0	-165.9	-176.7	-146.7	-400.9	-144.2	-155.0	-125.0	-379.2
	**2**	-203.8	-15.9	56.6	-246.2	-248.6	-192.0	-411.7	-224.7	-227.1	-170.5	-390.2
	**3**	-182.0	-23.8	103.2	-316.0	-311.0	-207.8	-413.6	-296.8	-291.8	-188.6	-394.5
	**4**	-159.0	-31.7	133.3	-376.3	-365.3	-232.0	-422.7	-360.5	-349.4	-216.2	-406.9
	**5**	-151.9	-39.6	177.2	-429.0	-411.3	-234.2	-425.8	-414.9	-397.3	-220.1	-411.7
	**6**	-164.6	-47.6	229.0	-480.9	-456.6	-227.6	-439.8	-471.9	-447.5	-218.6	-430.7
	**7**	-155.0	-55.5	267.0	-523.0	-492.3	-225.4	-435.8	-514.1	-483.4	-216.5	-426.9
	**8**	-148.6	-63.4	311.0	-566.0	-529.0	-218.1	-430.1	-559.9	-522.9	-212.0	-424.0
	**9**	-150.8	-71.3	358.7	-608.6	-565.3	-206.6	-428.8	-605.6	-562.3	-203.6	-425.8
	**10**	-148.7	-79.3	396.4	-643.2	-595.8	-199.5	-427.4	-638.0	-590.5	-194.2	-422.2
	**0**							-375.2				-375.2

Table S3 cont….

Anion	n^e	ΔΔG_{solv}	SScorr	-TΔS	DLPNO-SCS-MP2				DLPNO-CCSD(T)-F12			
					Δ$E_{bind,gp}$	Δ$H_{bind,gp}$	Δ$G_{bind,gp}$	ΔG_{solv}(X^-)	Δ$E_{bind,gp}$	Δ$H_{bind,gp}$	Δ$G_{bind,gp}$	ΔG_{solv}(X^-)
FHF^-	1	-228.3	-7.9	30.7	-61.9	-59.6	-28.9	-265.2	-67.9	-65.6	-34.9	-271.2
	2	-197.4	-15.9	62.0	-118.6	-111.6	-49.6	-262.8	-127.2	-120.1	-58.1	-271.3
	3	-185.8	-23.8	101.0	-169.1	-156.5	-55.5	-265.0	-179.0	-166.4	-65.4	-275.0
	4	-168.4	-31.7	137.1	-215.1	-195.5	-58.5	-258.5	-226.6	-207.0	-69.9	-270.0
	5	-169.5	-39.6	186.3	-256.3	-230.4	-44.2	-253.3	-265.7	-239.9	-53.6	-262.8
	6	-162.5	-47.6	227.2	-300.9	-268.9	-41.7	-251.8	-310.3	-278.3	-51.1	-261.2
	7	-155.9	-55.5	268.4	-343.8	-306.9	-38.6	-249.9	-353.6	-316.7	-48.3	-259.7
	8	-156.2	-63.4	313.2	-383.4	-344.1	-30.9	-250.5	-393.2	-353.8	-40.7	-260.2
	9	-148.5	-71.3	347.7	-425.0	-380.0	-32.3	-252.2	-434.5	-389.6	-41.9	-261.8
	10	-141.6	-79.3	394.2	-464.4	-413.6	-19.5	-240.3	-473.2	-422.4	-28.3	-249.1
	11	-149.2	-87.2	441.6	-504.9	-449.0	-7.4	-243.7				
	12	-134.5	-95.1	482.6	-540.8	-479.1	3.4	-226.2				
	13	-150.2	-103.0	531.7	-580.9	-514.1	17.6	-235.6				
	14	-137.2	-111.0	564.5	-612.1	-540.1	24.3	-223.9				
	0							-263.7				-263.7
F^-	1	-246.3	-7.9	30.0	-82.4	-93.2	-63.2	-317.4	-93.3	-104.0	-74.0	-328.3
	2	-203.8	-15.9	56.6	-159.7	-162.1	-105.5	-325.2	-173.3	-175.7	-119.1	-338.8
	3	-182.0	-23.8	103.2	-225.8	-220.8	-117.6	-323.5	-240.9	-235.9	-132.7	-338.5
	4	-159.0	-31.7	133.3	-283.7	-272.6	-139.3	-330.1	-301.0	-289.9	-156.7	-347.4
	5	-151.9	-39.6	177.2	-335.2	-317.5	-140.4	-332.0	-353.1	-335.4	-158.3	-349.8
	6	-164.6	-47.6	229.0	-385.8	-361.4	-132.5	-344.6	-403.0	-378.7	-149.7	-361.9
	7	-155.0	-55.5	267.0	-430.7	-400.1	-133.1	-343.6	-446.8	-416.1	-149.2	-359.6
	8	-148.6	-63.4	311.0	-475.1	-438.1	-127.2	-339.2	-489.9	-452.9	-142.0	-354.0
	9	-150.8	-71.3	358.7	-519.1	-475.7	-117.0	-339.2	-530.9	-487.5	-128.8	-351.0
	10	-148.7	-79.3	396.4	-549.7	-502.2	-105.9	-333.9	-562.3	-514.9	-118.6	-346.6
	0							-375.2				-375.2

Table S3 cont….

Anion	*n*[e]	$\Delta\Delta G_{solv}$	SScorr	-TΔS	BP86-D3(BJ) $\Delta E_{bind,gp}$	BP86-D3(BJ) $\Delta H_{bind,gp}$	BP86-D3(BJ) $\Delta G_{bind,gp}$	BP86-D3(BJ) $\Delta G_{solv}(X^{-})$	ωB97M-V $\Delta E_{bind,gp}$	ωB97M-V $\Delta H_{bind,gp}$	ωB97M-V $\Delta G_{bind,gp}$	ωB97M-V $\Delta G_{solv}(X^{-})$
ClF_2^-	1	-215.3	-7.9	30.3	-65.5	-62.8	-32.5	-255.7	-65.6	-62.9	-32.6	-255.8
	2	-188.1	-15.9	57.7	-119.5	-112.1	-54.4	-258.3	-122.5	-115.1	-57.4	-261.4
	3	-188.6	-23.8	110.8	-167.9	-154.8	-44.0	-256.4	-169.5	-156.3	-45.6	-257.9
	4	-173.8	-31.7	148.5	-216.9	-198.4	-49.9	-255.4	-219.3	-200.8	-52.3	-257.8
	5	-169.1	-39.6	186.8	-258.9	-234.8	-48.1	-256.7	-263.6	-239.5	-52.8	-261.5
	6	-171.2	-47.6	231.0	-300.7	-271.0	-40.1	-258.8	-308.3	-278.6	-47.7	-266.4
	7	-157.5	-55.5	271.0	-342.8	-307.8	-36.8	-249.8	-348.5	-313.6	-42.6	-255.6
	8	-155.3	-63.4	312.5	-385.0	-344.6	-32.2	-250.9	-396.9	-356.6	-44.1	-262.8
	9	-157.2	-71.3	355.9	-427.6	-382.1	-26.2	-254.7	-438.6	-393.1	-37.3	-265.8
	10	-155.2	-79.3	407.9	-466.8	-418.2	-10.3	-244.8	-483.3	-434.8	-26.8	-261.3
	11	-145.8	-87.2	433.9	-507.5	-451.2	-17.3	-250.2	-524.8	-468.5	-34.6	-267.6
	12	-148.1	-95.1	482.0	-550.5	-488.7	-6.7	-249.9	-565.5	-503.8	-21.8	-264.9
	13	-148.3	-103.0	530.3	-588.0	-520.9	9.3	-242.0	-607.4	-540.3	-10.1	-261.4
	14	-150.2	-111.0	571.4	-619.6	-547.0	24.3	-236.8	-643.3	-570.7	0.6	-260.5
	0							-240.9				-240.9
ClF_4^-	1	-201.7	-7.9	34.4	-57.0	-52.3	-17.9	-227.5	-59.9	-55.3	-20.9	-230.5
	2	-186.5	-15.9	65.1	-105.9	-96.3	-31.2	-233.5	-112.8	-103.2	-38.2	-240.5
	3	-175.5	-23.8	107.4	-147.9	-132.9	-25.4	-224.7	-155.8	-140.8	-33.3	-232.6
	4	-171.4	-31.7	153.0	-192.2	-174.4	-21.4	-224.5	-203.0	-185.1	-32.1	-235.3
	5	-163.2	-39.6	185.6	-230.2	-204.5	-18.9	-221.7	-242.8	-217.2	-31.6	-234.4
	6	-165.6	-47.6	227.4	-269.9	-239.0	-11.6	-224.8	-287.9	-257.0	-29.6	-242.7
	7	-161.5	-55.5	268.2	-311.1	-274.7	-6.6	-223.5	-328.6	-292.2	-24.1	-241.0
	8	-157.5	-63.4	311.2	-349.4	-307.6	3.5	-217.4	-369.1	-327.3	-16.1	-237.0
	9	-155.6	-71.3	348.9	-388.5	-341.5	7.4	-219.5	-409.4	-362.3	-13.5	-240.4
	10	-154.7	-79.3	393.6	-427.6	-375.3	18.3	-215.6	-454.6	-402.2	-8.7	-242.6
	11	-152.2	-87.2	426.9	-468.3	-411.0	15.9	-223.5	-502.8	-445.6	-18.7	-258.1
	12	-146.0	-95.1	479.2	-512.8	-449.2	30.0	-211.2	-543.8	-480.2	-1.0	-242.2
	13	-149.3	-103.0	520.7	-547.9	-479.6	41.1	-211.2	-581.5	-513.2	7.5	-244.8
	14	-148.1	-111.0	563.9	-582.8	-509.0	54.8	-204.3	-616.0	-542.1	21.7	-237.4
	0							-223.6				-223.6

Table S3. (Continued)

Anion	n^e	ΔΔG_{solv}	SScorr	-TΔS	DLPNO-SCS-MP2 Δ$E_{bind,gp}$	Δ$H_{bind,gp}$	Δ$G_{bind,gp}$	Δ$G_{solv}(X^-)$	DLPNO-CCSD(T)-F12 Δ$E_{bind,gp}$	Δ$H_{bind,gp}$	Δ$G_{bind,gp}$	Δ$G_{solv}(X^-)$
ClF_2^-	1	-215.3	-7.9	30.3	-54.2	-51.4	-21.1	-244.4	-57.6	-54.9	-24.6	-247.9
	2	-188.1	-15.9	57.7	-103.9	-96.4	-38.8	-242.7	-109.8	-102.3	-44.7	-248.6
	3	-188.6	-23.8	110.8	-147.4	-134.3	-23.5	-235.8	-153.8	-140.6	-29.8	-242.2
	4	-173.8	-31.7	148.5	-193.8	-175.3	-26.8	-232.3	-200.4	-181.9	-33.4	-238.9
	5	-169.1	-39.6	186.8	-235.3	-211.3	-24.5	-233.2	-241.9	-217.8	-31.1	-239.8
	6	-171.2	-47.6	231.0	-277.3	-247.7	-16.7	-235.4	-283.9	-254.2	-23.3	-242.0
	7	-157.5	-55.5	271.0	-314.5	-279.5	-8.6	-221.5	-322.8	-287.9	-16.9	-229.9
	8	-155.3	-63.4	312.5	-361.5	-321.2	-8.7	-227.4	-367.4	-327.1	-14.6	-233.3
	9	-157.2	-71.3	355.9	-401.1	-355.6	0.3	-228.2	-405.3	-359.8	-4.0	-232.5
	10	-155.2	-79.3	407.9	-443.6	-395.0	12.9	-221.6	-446.6	-398.1	9.8	-224.6
	11	-145.8	-87.2	433.9	-484.0	-427.7	6.2	-226.8				
	12	-148.1	-95.1	482.0	-523.5	-461.7	20.3	-222.9				
	13	-148.3	-103.0	530.3	-562.8	-495.8	34.5	-216.9				
	14	-150.2	-111.0	571.4	-596.5	-523.9	47.4	-213.7				
	0							-240.9				-240.9
ClF_4^-	1	-201.7	-7.9	34.4	-50.4	-45.7	-11.3	-220.9	-52.8	-48.1	-13.7	-223.4
	2	-186.5	-15.9	65.1	-95.8	-86.2	-21.2	-223.5	-99.8	-90.2	-25.2	-227.5
	3	-175.5	-23.8	107.4	-136.5	-121.4	-14.0	-213.3	-139.6	-124.6	-17.1	-216.4
	4	-171.4	-31.7	153.0	-181.5	-163.6	-10.7	-213.8	-182.0	-164.2	-11.2	-214.3
	5	-163.2	-39.6	185.6	-216.8	-191.1	-5.6	-208.4	-222.1	-196.4	-10.9	-213.7
	6	-165.6	-47.6	227.4	-258.8	-227.8	-0.4	-213.6	-262.0	-231.0	-3.6	-216.8
	7	-161.5	-55.5	268.2	-299.5	-263.1	5.0	-211.9	-300.8	-264.4	3.7	-213.3
	8	-157.5	-63.4	311.2	-337.0	-295.2	15.9	-205.0	-340.8	-299.0	12.2	-208.7
	9	-155.6	-71.3	348.9	-375.5	-328.5	20.3	-206.6	-375.9	-328.9	19.9	-207.0
	10	-154.7	-79.3	393.6	-421.1	-368.7	24.9	-209.0				
	11	-152.2	-87.2	426.9	-463.7	-406.4	20.5	-218.9				
	12	-146.0	-95.1	479.2	-505.4	-441.7	37.4	-203.7				
	13	-149.3	-103.0	520.7	-542.0	-473.7	47.0	-205.3				
	14	-148.1	-111.0	563.9	-576.0	-502.2	61.6	-197.5				
	0							-223.6				-223.6

Table S3 cont….

[a] Total electronic energies at the indicated levels were obtained by corresponding single-point calculations at MARIJ-BP86-D3(BJ)/def2-TZVPP gas-phase optimized structures.
[b] Thermal and entropic contributions at standard state conditions (298.15 K, 0.1 MPa) were evaluated within the ideal gas and rigid rotor – harmonic oscillator approximation based on harmonic vibrational frequency calculations at MARIJ-BP86-D3(BJ)/def2-TZVPP level.
[c] $\Delta\Delta G_{solv} = \Delta G_{solv}(X^{-}(MeCN)_{n}) - n\Delta G_{solv}(MeCN)$; COSMO-RS solvation free energies were calculated by single-point calculations at MARIJ-BP86/def2-TZVPD[/COSMO(MeCN)] level at MARIJ-BP86-D3(BJ)/def2-TZVPP[/COSMO(MeCN)] optimized structures.
[d] $SScorr = - nRT\ln(V_m)$; $V_m = 24.46$ mol L^{-1}.
[e] Index 0 indicates the COSMO-RS solvation free energy of X^- calculated without the inclusion of explicit solvent molecules, see the Computational Details

Figure S3. Comparison of gas-phase binding free energies ($\Delta G_{bind,gp}$) of anions X^- in acetonitrile (X^- = ClF_2^-, ClF_4^-, FHF^-, F^-) using either $(MeCN)_n$ clusters (cluster cycle) or n distinct MeCN molecules (monomer cycle), calculated at DLPNO-SCS-MP2/aug-cc-pVTZ level ("no embedding"). All energies are given in kJ mol^{-1}. Fitting curves are included only for the purpose of guiding the eye.

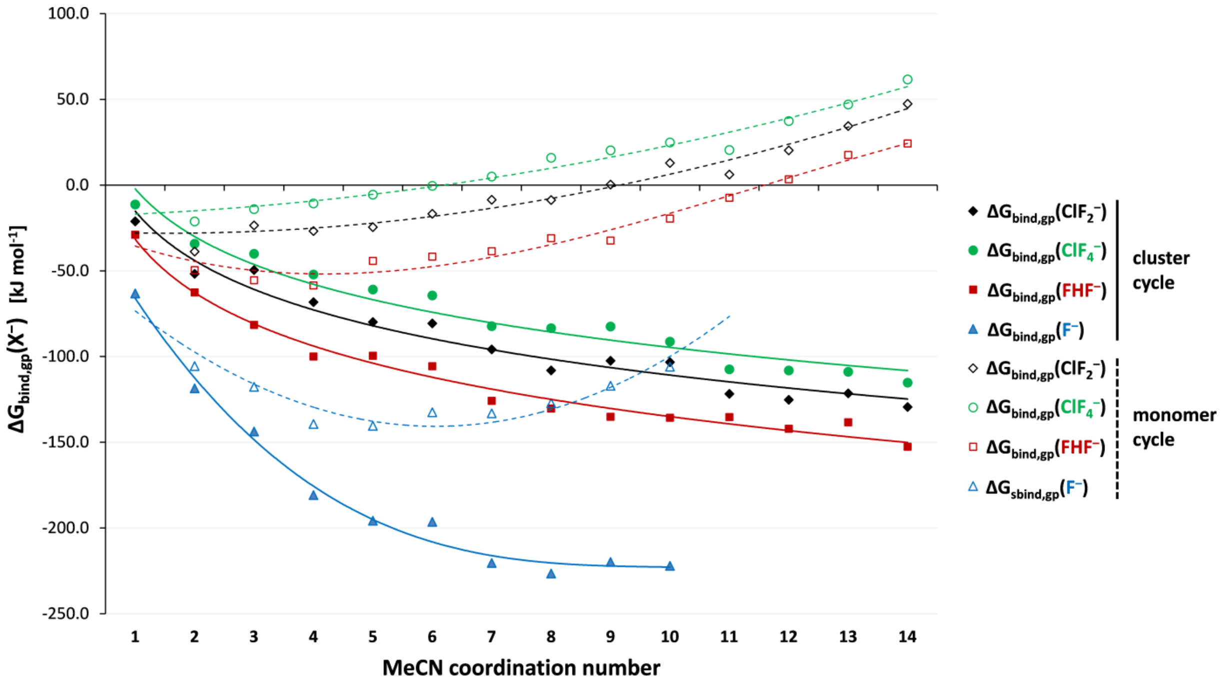

Figure S4. Comparison of solvation free energies (ΔG_{solv}) of anions X^- in acetonitrile (X^- = ClF_2^-, ClF_4^-, FHF^-, F^-) using either explicit $(MeCN)_n$ clusters (cluster cycle) or *n* distinct MeCN molecules (monomer cycle), calculated at DLPNO-SCS-MP2/aug-cc-pVTZ/COSMO-RS level ("COSMO-RS embedding"). All energies are given in kJ mol^{-1}. Fitting curves are included only for the purpose of guiding the eye.

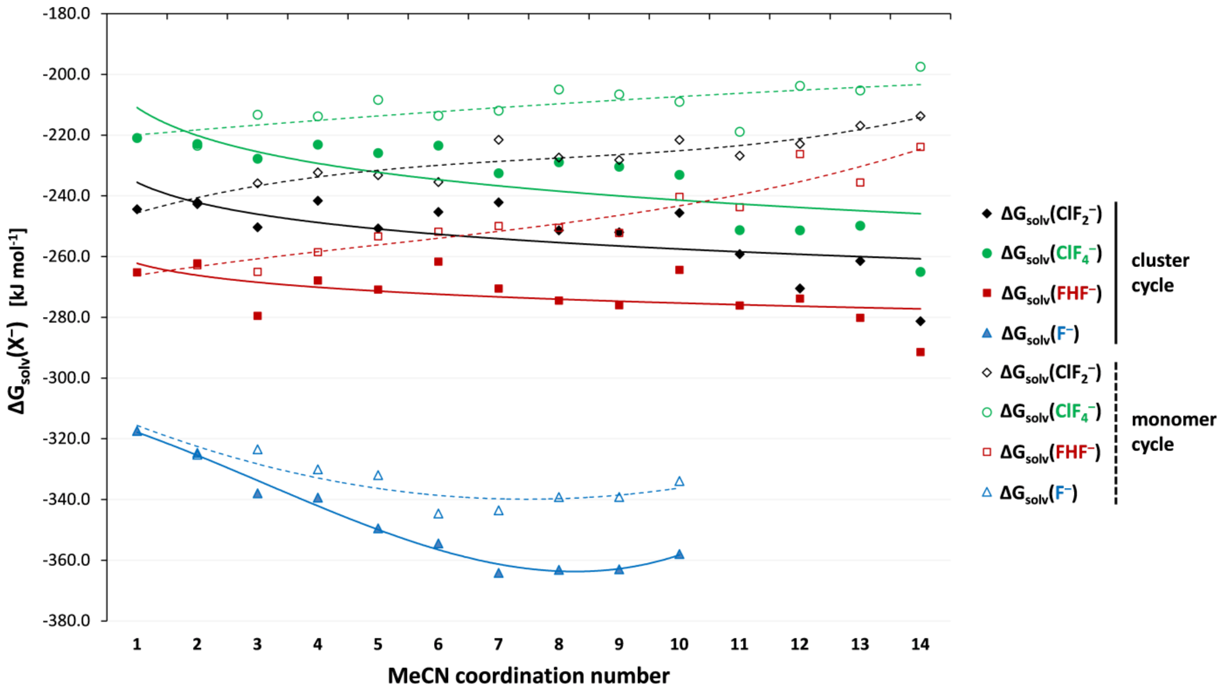

Figure S5. Comparison of computed electronic gas-phase binding energies ($\Delta E_{bind,gp}$) of F^- with $(MeCN)_n$ clusters ($n = 1 - 10$) at different computational levels (no embedding). All energies are given in kJ mol^{-1}.

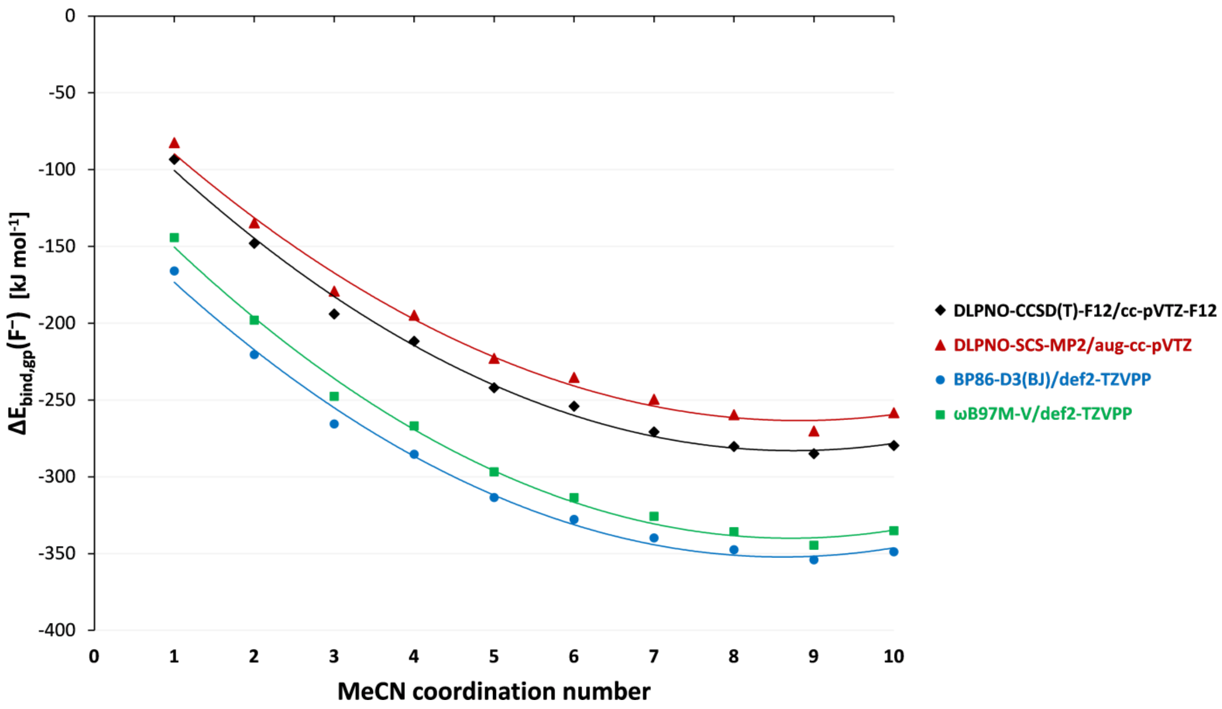

Figure S6. Comparison of solvation free energies (ΔG_{solv}) of anions X^- in acetonitrile (X^- = ClF_2^-, ClF_4^-, FHF^-, F^-), obtained by using either explicit $(MeCN)_n$ clusters (cluster cycle; DLPNO-SCS-MP2/aug-cc-pVTZ/COSMO-RS; data points with solid fitting curves) or pure COSMO-RS computations without explicit solvent molecules (BP86/def2-TZVPD[/COSMO(MeCN)]; horizontal dashed lines). All energies are given in kJ mol^{-1}. Fitting curves are included only for the purpose of guiding the eye.

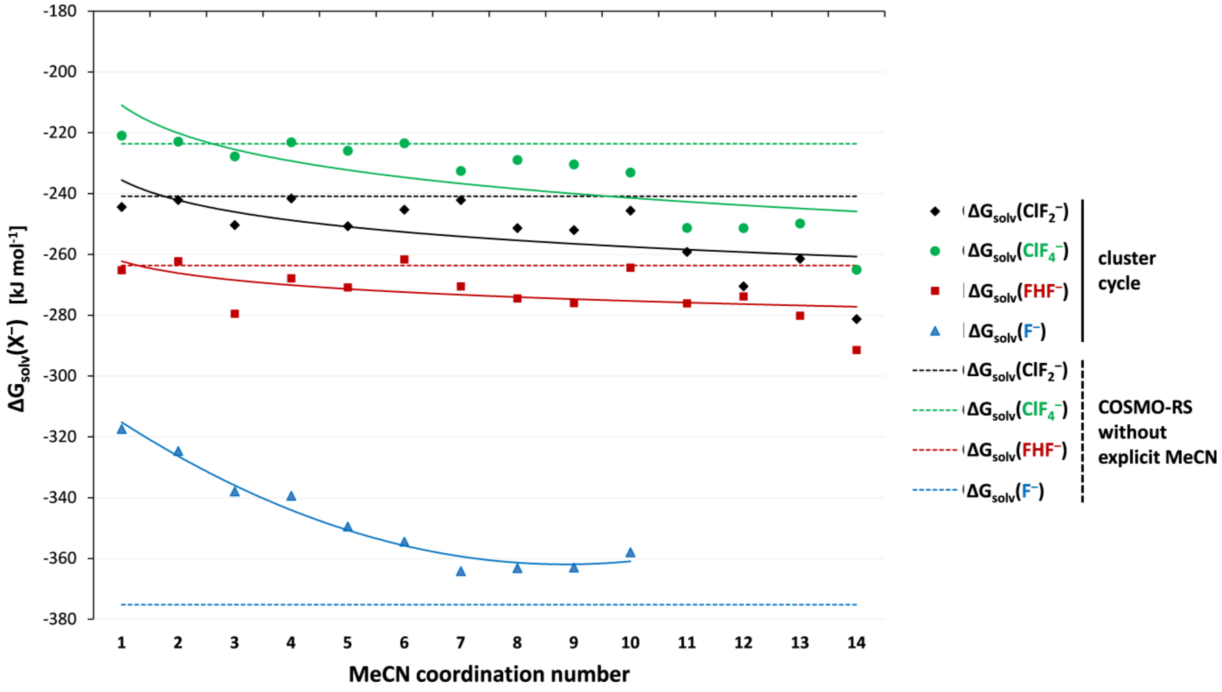

S4. NMR chemical shifts in MeCN

Table S4. Computed ^{19}F-NMR shifts in ppm for microsolvated fluoride-like anions in MeCN with increasing cluster size using different functionals.[a]

anion	n	exp. Shift	BHLYP	cLH12sir	cLH12sif	cLH20t
F^-	0	-74	-293.5	-293.3	-290.1	-295.8
	1		-194.0	-199.8	-198.0	-200.3
	2		-151.8	-160.4	-159.3	-158.6
	3		-117.3	-128.5	-128.0	-125.0
	4		-93.2	-104.7	-104.7	-99.8
	5		-71.5	-83.1	-83.5	-77.3
	6		-37.0	-50.6	-51.6	-43.3
	7		-44.1	-54.8	-55.9	-46.1
	8		-57.3	-65.9	-66.9	-57.0
	9		-77.5	-82.8	-83.7	-75.4
	10		-58.2	-66.3	-67.2	-57.3
	11		-58.6	-66.1	-67.1	-57.5
	n					
FHF^-	0	145-149	-211.6	-218.7	-216.5	-219.1
	1		-195.5	-203.7	-201.7	-203.2
	2		-182.9	-191.6	-189.9	-190.4
	3		-171.5	-180.8	-179.3	-178.7
	4		-164.3	-174.0	-172.7	-171.4
	5		-154.2	-164.6	-163.4	-160.8
	6		-145.5	-156.1	-155.1	-151.8
	7		-139.2	-150.0	-149.1	-145.0
	8		-130.4	-141.7	-140.9	-136.0
	9		-125.3	-136.9	-136.3	-130.7
	10		-124.1	-134.5	-133.9	-128.3
	11		-124.3	-135.2	-134.6	-128.1
	12		-129.7	-140.2	-139.6	-133.2
	n					
ClF_2^-	0	-125	-206.3	-167.9	-168.8	-174.7
	1		-193.2	-156.5	-157.4	-162.0
	2		-184.6	-149.1	-150.2	-153.4
	3		-173.9	-139.0	-140.3	-143.3
	4		-166.7	-132.2	-133.7	-136.0
	5		-160.7	-126.5	-128.0	-128.9
	6		-154.0	-120.6	-122.2	-122.0
	7		-147.8	-115.4	-117.2	-116.4
	8		-147.1	-115.4	-117.0	-114.7
	9		-137.3	-105.4	-107.0	-106.3
	10		-130.2	-98.3	-100.0	-98.1
	11		-126.7	-95.1	-96.7	-94.3
	12		-125.5	-93.4	-95.0	-93.6
	n					
ClF_4^-	0	67	67.8	67.8	65.4	69.0
	1		73.0	72.2	69.6	73.8
	2		76.2	75.0	72.4	77.4
	3		83.8	83.1	80.4	86.2
	4		78.0	77.7	75.2	81.1
	5		76.2	75.8	73.3	79.0
	6		77.7	77.7	75.1	81.2
	7		78.2	78.5	75.9	82.6
	8		80.7	80.2	77.6	83.9
	9		87.0	86.0	83.3	89.9
	10		81.8	81.8	79.3	85.5
	11		82.8	83.6	81.0	88.3
	12		83.5	83.2	80.5	87.1

[a]MARIJ-GIAO-DFT/pcSseg-3/COSMO//MARIJ-D3(BJ)-BP86/def2-TZVPP/COSMO level. Relative to a computed $CFCl_3$(l) reference shielding of 187.2 ppm (BHLYP), 186.3 ppm (cLH12ct-SsirPW92), 189.5 ppm (cLH12ct-SsifPW92), 184.6 ppm (cLH20t). Experimental data from Refs. S1, S2, S3. cLH12sir and cLH12sif abbreviate cLH12ct-SsirPW92 and cLH12ct-SsifPW92, respectively. The "c" for the LH functionals denotes inclusion of current-density response.

Table S5. Computed ^{19}F-NMR chemical-shift anisotropies in ppm for microsolvated fluoride-like anions in MeCN with increasing cluster size using different functionals.[a]

anion	n	BHLYP	cLH12sir[a]	cLH12sif[a]	cLH20t
F^-	0	0.0	0.0	0.0	0.0
	1	150.3	142.0	140.1	144.5
	2	85.6	82.2	81.4	84.0
	3	3.9	3.9	4.2	3.6
	4	74.8	71.9	71.0	72.9
	5	53.4	52.8	51.9	53.1
	6	41.7	35.3	35.2	39.6
	7	25.5	21.3	20.9	24.3
	8	18.6	14.5	14.2	17.6
	9	18.1	19.5	19.2	18.2
	10	14.6	13.5	13.1	14.4
	11	15.6	15.2	14.8	15.5
	n				
FHF^-	0	128.5	117.5	116.2	120.8
	1	112.6	103.6	102.5	105.9
	2	98.2	90.1	89.3	91.7
	3	90.6	83.7	82.9	85.0
	4	104.3	95.9	94.8	97.9
	5	101.5	93.7	92.9	95.0
	6	86.4	80.4	79.7	80.4
	7	85.7	79.6	79.1	79.4
	8	79.7	73.0	72.7	72.3
	9	78.9	72.1	71.8	71.6
	10	78.2	72.5	72.3	70.6
	11	89.4	81.9	81.5	82.9
	12	91.6	84.8	84.3	84.9
	n				
ClF_2^-	0	145.4	202.6	196.6	196.3
	1	139.3	195.8	190.1	189.8
	2	131.0	187.3	181.6	182.1
	3	126.4	182.5	177.2	176.1
	4	120.5	177.7	172.4	170.9
	5	116.2	173.8	168.6	168.1
	6	111.5	167.5	162.4	162.7
	7	108.5	164.1	159.1	159.1
	8	123.4	175.9	171.0	172.8
	9	125.3	180.3	175.7	173.1
	10	122.2	177.9	173.4	170.6
	11	132.6	187.7	183.3	180.7
	12	130.1	185.6	181.2	178.0
	n				
ClF_4^-	0	445.6	430.8	426.5	434.9
	1	441.4	426.6	422.4	430.5
	2	435.8	421.4	417.3	425.0
	3	428.6	415.2	411.3	419.1
	4	426.0	412.9	409.0	417.0
	5	424.8	411.5	407.7	415.2
	6	419.2	406.7	403.0	410.5
	7	416.2	404.1	400.4	407.8
	8	414.1	402.3	398.7	404.8
	9	413.4	401.4	397.7	403.6
	10	408.5	397.2	393.8	399.1
	11	407.5	396.4	393.0	399.3
	12	395.0	385.2	381.9	386.1

[a]MARIJ-GIAO-DFT/pcSseg-3/COSMO//MARIJ-D3(BJ)-BP86/def2-TZVPP/COSMO level. Relative to a computed $CFCl_3$(l) reference shielding of 187.2 ppm (BHLYP), 186.3 ppm (cLH12ct-SsirPW92), 189.5 ppm (cLH12ct-SsifPW92), 184.6 ppm (cLH20t). cLH12sir and cLH12sif abbreviate cLH12ct-SsirPW92 and cLH12ct-SsifPW92, respectively. The "c" for the LH functionals denotes inclusion of current-density response.

S5. Thermochemical data in water

Table S6. Computed gas-phase binding energies ($\Delta E_{bind,gp}$), binding enthalpies ($\Delta H_{bind,gp}$), free binding energies ($\Delta G_{bind,gp}$), and final solvation free energies ΔG_{solv} of F^- in water, using explicit $(H_2O)_n$ clusters (cluster cycle) or *n* distinct H_2O molecules (monomer cycle, *n* = 1 − 12), at DFT (BP86-D3(BJ)/def2-TZVPP, ωB97M-V/def2-TZVPP), DLPNO-SCS-MP2/aug-cc-pVTZ, and DLPNO-CCSD(T)-F12/cc-pVTZ-F12 levels.[a] Thermal and entropic contributions have been evaluated at standard state conditions (298.15 K, 0.1 MPa).[b] $\Delta\Delta G_{solv}$ is the bulk solvent free energy change obtained from COSMO-RS computations,[c] SScorr is the standard state correction,[d] and $-T\Delta S$ is the entropic contribution term.[b] All energies are given in kJ mol^{-1}.

					BP86-D3(BJ)				ωB97M-V			
F^-	*n*[e]	$\Delta\Delta G_{solv}$	SScorr	$-T\Delta S$	$\Delta E_{bind,gp}$	$\Delta H_{bind,gp}$	$\Delta G_{bind,gp}$	ΔG_{solv}	$\Delta E_{bind,gp}$	$\Delta H_{bind,gp}$	$\Delta G_{bind,gp}$	ΔG_{solv}
$(H_2O)_n$	1	-309.5	-7.9	57.1	-150.2	-154.1	-124.4	-441.8	-140.7	-144.6	-114.9	-432.3
	2	-263.0	-7.9	52.1	-233.1	-234.7	-200.0	-470.9	-223.3	-224.8	-190.2	-461.1
	3	-258.4	-7.9	60.5	-260.6	-261.5	-235.2	-501.5	-257.0	-257.9	-231.6	-497.9
	4	-212.8	-7.9	52.8	-290.0	-288.3	-254.3	-475.1	-289.2	-287.6	-253.5	-474.3
	5	-180.1	-7.9	44.3	-315.9	-310.7	-268.2	-456.2	-321.0	-315.8	-273.3	-461.3
	6	-185.4	-7.9	51.7	-340.8	-336.7	-301.7	-494.9	-341.1	-337.1	-302.0	-495.2
	7	-159.7	-7.9	49.1	-347.8	-343.5	-305.8	-473.4	-347.4	-343.0	-305.3	-472.9
	8	-189.7	-7.9	58.2	-332.1	-329.3	-300.7	-498.3	-334.4	-331.7	-303.1	-500.7
	9	-175.7	-7.9	51.2	-353.9	-348.8	-313.2	-496.8	-357.2	-352.1	-316.5	-500.1
	10	-164.2	-7.9	56.2	-350.9	-344.8	-314.2	-486.3	-362.5	-356.4	-325.8	-497.9
	11	-134.7	-7.9	48.8	-388.0	-381.9	-343.9	-486.5	-389.1	-383.0	-344.9	-487.6
	12	-164.0	-7.9	56.5	-374.4	-370.8	-340.5	-512.4	-374.1	-370.5	-340.2	-512.2
	0							-437.2				-437.2
n H_2O	1	-309.5	-7.9	57.1	-150.2	-154.1	-124.4	-441.8	-140.7	-144.6	-114.9	-432.3
	2	-292.5	-15.9	22.7	-256.3	-251.1	-186.9	-495.3	-246.7	-241.5	-177.3	-485.7
	3	-270.1	-23.8	-17.5	-338.4	-324.4	-220.0	-513.9	-331.4	-317.3	-213.0	-506.9
	4	-230.6	-31.7	-68.2	-428.1	-405.0	-250.0	-512.3	-416.1	-392.9	-237.9	-500.2
	5	-208.1	-39.6	-113.0	-496.3	-463.7	-264.0	-511.6	-486.2	-453.6	-253.8	-501.5
	6	-199.4	-47.6	-157.7	-572.0	-531.7	-287.2	-534.1	-557.2	-516.9	-272.4	-519.3
	7	-183.4	-55.5	-204.8	-636.6	-589.0	-297.4	-536.3	-617.2	-569.6	-278.0	-516.9
	8	-186.6	-63.4	-249.5	-700.5	-646.4	-310.1	-560.1	-674.3	-620.1	-283.9	-533.9
	9	-184.9	-71.3	-297.4	-768.2	-705.9	-321.7	-577.9	-737.9	-675.6	-291.4	-547.6
	10	-170.5	-79.3	-339.7	-822.4	-752.1	-325.7	-575.4	-795.8	-725.5	-299.0	-548.8
	11	-137.6	-87.2	-391.7	-905.8	-828.9	-350.4	-575.2	-864.8	-787.9	-309.4	-534.2
	12	-135.4	-95.1	-432.1	-954.6	-868.9	-350.0	-580.5	-915.2	-829.4	-310.5	-541.1
	0							-437.2				-437.2

Table S6. (Continued)

					DLPNO-SCS-MP2				DLPNO-CCSD(T)-F12			
**F^-**	***n*[e]**	**$\Delta\Delta G_{solv}$**	**SScorr**	**$-T\Delta S$**	**$\Delta E_{bind,gp}$**	**$\Delta H_{bind,gp}$**	**$\Delta G_{bind,gp}$**	**ΔG_{solv}**	**$\Delta E_{bind,gp}$**	**$\Delta H_{bind,gp}$**	**$\Delta G_{bind,gp}$**	**ΔG_{solv}**
**$(H_2O)_n$**	**1**	-309.5	-7.9	57.1	-109.1	-113.0	-83.3	-400.7	-113.9	-117.7	-88.0	-405.5
	**2**	-263.0	-7.9	52.1	-174.6	-176.1	-141.4	-412.4	-183.0	-184.6	-149.9	-420.8
	**3**	-258.4	-7.9	60.5	-206.4	-207.3	-181.0	-447.3	-214.0	-214.9	-188.6	-454.9
	**4**	-212.8	-7.9	52.8	-228.7	-227.1	-193.1	-413.8	-239.3	-237.6	-203.6	-424.4
	**5**	-180.1	-7.9	44.3	-253.2	-248.0	-205.5	-393.5	-266.2	-261.0	-218.5	-406.5
	**6**	-185.4	-7.9	51.7	-279.0	-275.0	-239.9	-433.2	-289.8	-285.7	-250.6	-443.9
	**7**	-159.7	-7.9	49.1	-285.3	-280.9	-243.2	-410.9	-296.5	-292.1	-254.4	-422.0
	**8**	-189.7	-7.9	58.2	-276.1	-273.4	-244.8	-442.4	-285.7	-283.0	-254.4	-452.0
	**9**	-175.7	-7.9	51.2	-292.8	-287.7	-252.1	-435.7	-304.3	-299.2	-263.6	-447.2
	**10**	-164.2	-7.9	56.2	-296.3	-290.2	-259.6	-431.8	-308.2	-302.1	-271.5	-443.6
	**11**	-134.7	-7.9	48.8	-324.6	-318.5	-280.5	-423.2	-336.4	-330.3	-292.3	-435.0
	**12**	-164.0	-7.9	56.5	-313.9	-310.3	-280.0	-451.9	-323.9	-320.3	-290.0	-461.9
	**13**	-309.5	-7.9	57.1	-109.1	-113.0	-83.3	-400.7	-113.9	-117.7	-88.0	-405.5
	**14**	-263.0	-7.9	52.1	-174.6	-176.1	-141.4	-412.4	-183.0	-184.6	-149.9	-420.8
	**0**							-437.2				-437.2
***n* H_2O**	**1**	-309.5	-7.9	57.1	-109.1	-113.0	-83.3	-400.7	-113.9	-117.7	-88.0	-405.5
	**2**	-292.5	-15.9	22.7	-194.2	-189.0	-124.9	-433.3	-203.3	-198.1	-134.0	-442.3
	**3**	-270.1	-23.8	-17.5	-264.6	-250.5	-146.2	-440.1	-276.6	-262.6	-158.2	-452.1
	**4**	-230.6	-31.7	-68.2	-331.9	-308.8	-153.8	-416.1	-348.5	-325.3	-170.3	-432.6
	**5**	-208.1	-39.6	-113.0	-389.0	-356.4	-156.6	-404.3	-409.3	-376.8	-177.0	-424.7
	**6**	-199.4	-47.6	-157.7	-450.9	-410.6	-166.0	-413.0	-473.3	-433.0	-188.5	-435.4
	**7**	-183.4	-55.5	-204.8	-501.2	-453.6	-162.0	-400.8	-526.7	-479.1	-187.5	-426.3
	**8**	-186.6	-63.4	-249.5	-549.3	-495.2	-158.9	-408.9	-577.1	-523.0	-186.7	-436.7
	**9**	-184.9	-71.3	-297.4	-600.9	-538.7	-154.5	-410.7	-631.9	-569.7	-185.4	-441.6
	**10**	-170.5	-79.3	-339.7	-647.8	-577.5	-151.0	-400.8	-681.4	-611.1	-184.6	-434.4
	**0**							-437.2				-437.2

[a] Total electronic energies at the indicated levels were obtained by corresponding single-point calculations at MARIJ-BP86-D3(BJ)/def2-TZVPP gas-phase optimized structures.

[b] Thermal and entropic contributions at standard state conditions (298.15 K, 0.1 MPa) were evaluated within the ideal gas and rigid rotor – harmonic oscillator approximation based on harmonic vibrational frequency calculations at MARIJ-BP86-D3(BJ)/def2-TZVPP level.

[c] Cluster cycle: $\Delta\Delta G_{solv} = \Delta G_{solv}(X^-(MeCN)_n) - \Delta G_{solv}((MeCN)_n)$; monomer cycle: $\Delta\Delta G_{solv} = \Delta G_{solv}(X^-(MeCN)_n) - n\Delta G_{solv}(MeCN)$; COSMO-RS solvation free energies were calculated by single-point calculations at MARIJ-BP86/def2-TZVPD[/COSMO(MeCN)] level at MARIJ-BP86-D3(BJ)/def2-TZVPP[/COSMO(MeCN)] optimized structures.

[d] SScorr = $-nRT\ln(V_m)$; V_m = 24.46 mol L^{-1}.

[e] Index 0 indicates the COSMO-RS solvation free energy of F^- calculated without the inclusion of explicit H_2O solvent molecules, see the Computational Details.

S6. Dependence of energies and shifts on cluster structure for F- in MeCN

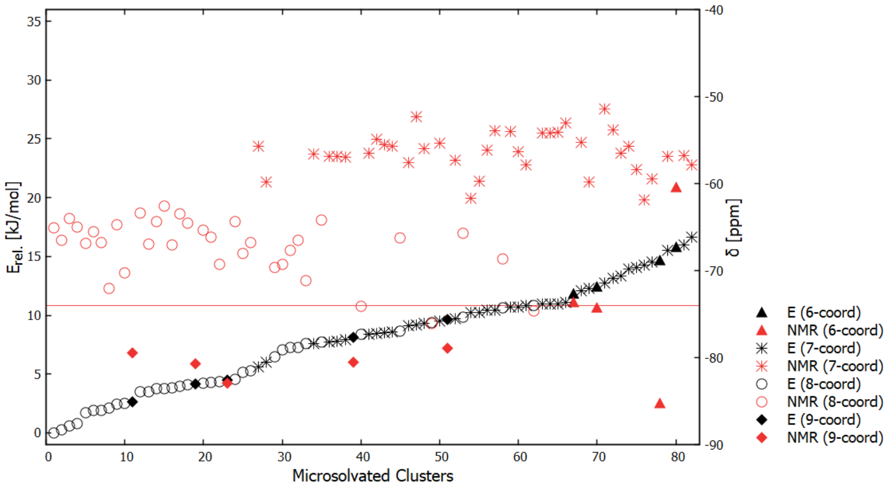

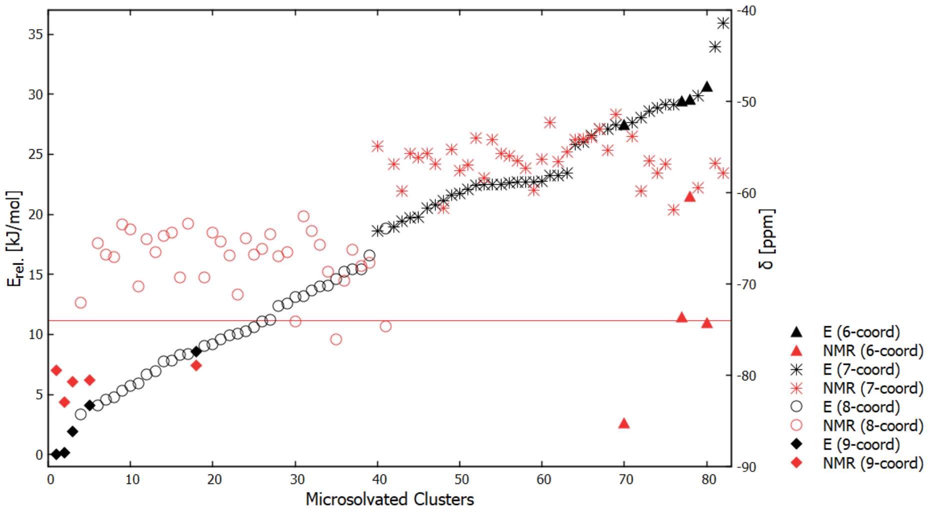

Figure S7. Plots of energies and ^{19}F NMR shifts for different microsolvated $F^-(MeCN)_n$ (n = 6, 7, 8) clusters obtained in BP86-D3/def2-TZVPP/COSMO optimizations following the xTB-based cluster generation. Energies are either given at BP86-D3/def2-TZVPP/COSMO level (top) or at LH12ct-SsirPW92-D3/pcSseg-3//BP86-D3/def2-TZVPP/COSMO level (bottom), shifts at cLH12ct-SsirPW92-D3/pcSseg-3 level.

S7. Further results for F^- in aqueous solution

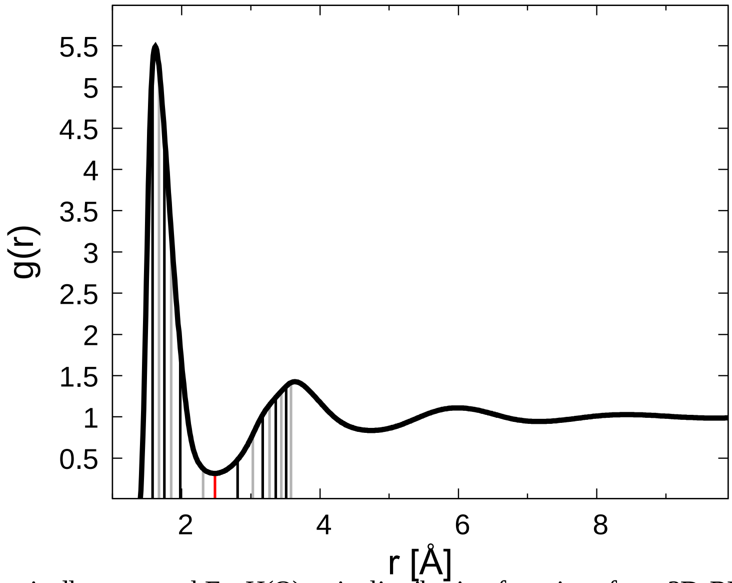

Figure S8. Spherically averaged F···H(O) pair distribution functions from 3D-RISM-SCF calculations of the fluoride ion in water. The vertical lines mark the positions, up to which g(r) integrates to an integer number of H sites, the red line marks the minimum.

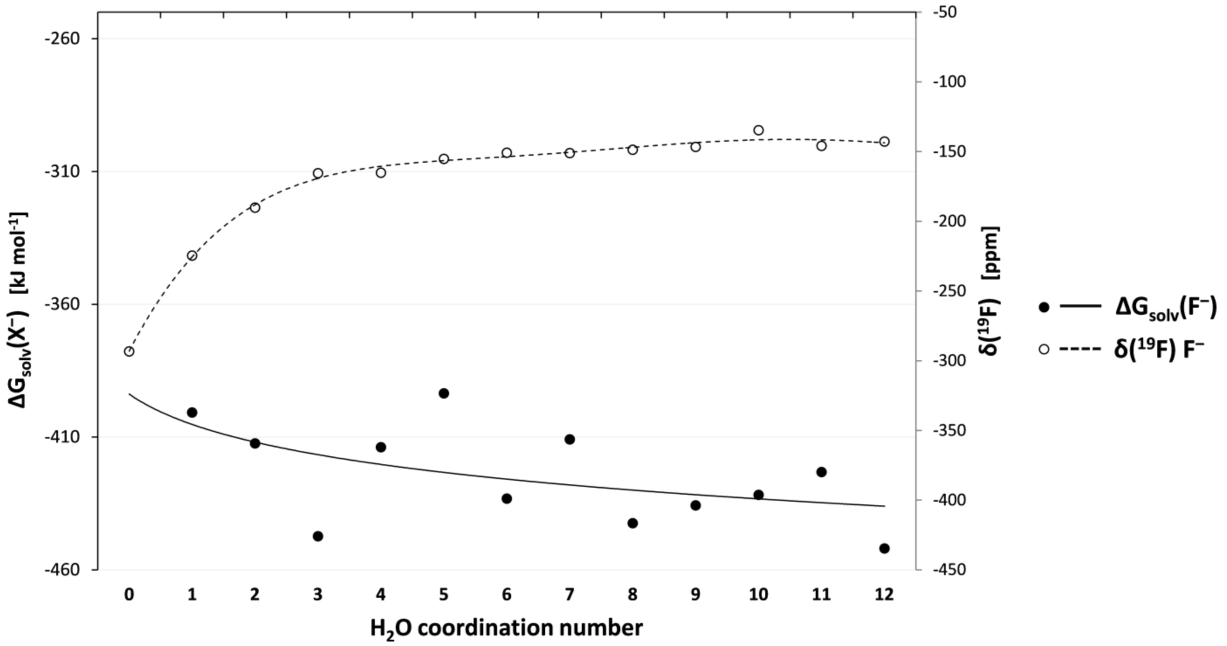

Figure S9. Comparison of computed solvation free energies (ΔG_{solv}) of F^- using explicit $(H_2O)_n$ clusters with COSMO-RS embedding (cluster cycle; $n = 1 - 12$, DLPNO-SCS-MP2/aug-cc-pVTZ/COSMO-RS//MARIJ-BP86-D3(BJ)/def2-TZVPP) and ^{19}F-NMR chemical shifts (GIAO-cLH12ct-SsirPW92/pcSseg-3/COSMO(H_2O)//MARIJ-BP86-D3(BJ)/def2-TZVPP/COSMO(H_2O). All energies are given in kJ mol^{-1}, δ(^{19}F) in ppm.

Table S7. Computed ^{19}F-NMR shifts in ppm for the microsolvated fluoride anion in H_2O with increasing cluster size using different functionals.[a]

anion	n	BHLYP	cLH12sir	cLH12sif	cLH20t
F^-	0	-293.5	-293.3	-290.2	-295.8
	1	-217.2	-224.6	-222.2	-225.1
	2	-179.2	-190.2	-188.4	-189.3
	3	-152.2	-165.5	-164.2	-163.0
	4	-153.4	-165.1	-163.8	-162.8
	5	-143.6	-155.2	-154.2	-151.7
	6	-138.1	-150.8	-149.9	-146.6
	7	-138.5	-151.1	-150.1	-146.3
	8	-135.8	-148.7	-147.8	-144.3
	9	-132.7	-146.6	-145.7	-142.3
	10	-120.8	-134.6	-134.1	-129.3
	11	-132.7	-145.8	-144.9	-141.6
	12	-129.1	-142.8	-141.9	-138.0

[a]MARIJ-GIAO-DFT/pcSseg-3/COSMO//MARIJ-D3(BJ)-BP86/def2-TZVPP/COSMO shifts relative to computed $CFCl_3$(l) reference shieldings of 187.2 ppm (BHLYP), 186.3 ppm (cLH12ct-SsirPW92), 189.5 ppm (cLH12ct-SsifPW92), 184.6 ppm (cLH20t). The “c” for the LH functionals denotes inclusion of current-density response. These data should be compared to an experimental shift of -119 ppm.[S1] cLH12sir and cLH12sif abbreviate cLH12ct-SsirPW92 and cLH12ct-SsifPW92, respectively.

S8. Analyses of solvent effects on NMR chemical shifts

Table S8. Contributions to the magnetic shielding tensor of the fluoride ion at (COSMO)-GIAO-MARIJ-cLH12ct-SsirPW92/pcSseg-3 level in the gas phase and using either 6 explicit water or 8 MeCN solvent molecules.[a]

F^-	contribution	σ_{11}	σ_{22}	σ_{33}	σ_{iso}
gas phase	diamagnetic	479.6	479.6	479.6	479.6
	pramagn. undist.	0.0	0.0	0.0	0.0
	paramagn. dist.	0.0	0.0	0.0	0.0
+6 H_2O	diamagnetic	481.5	481.5	475.3	479.4
(S_6 symmetry)	paramagn. undist.	2.3	2.3	8.4	4.3
	paramagn. dist.	-142.2	-142.2	-197.1	-160.5
+8 MeCN	diamagnetic	482.5	482.2	482.2	482.3
	paramagn. undist.	17.3	31.6	28.3	25.7
	paramagn. dist.	-255.1	-258.4	-253.8	-255.8

[a]GIAO-MARIJ-cLH12ct-SsirPW92/pcSseg-3/COSMO level.

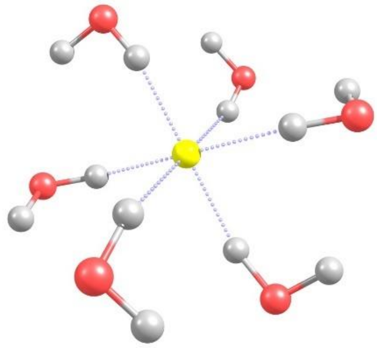

15

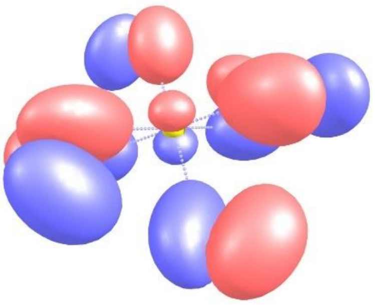

16

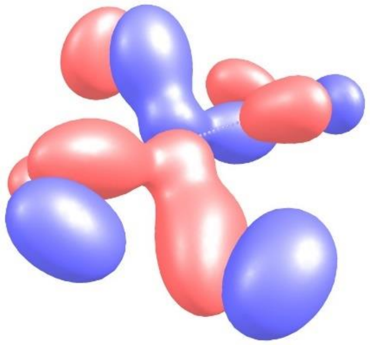

17

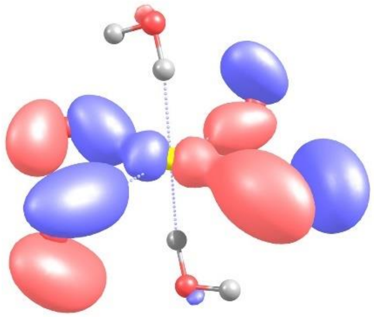

21

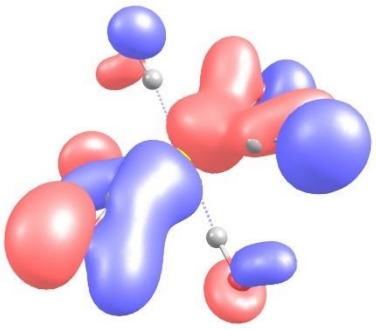

22

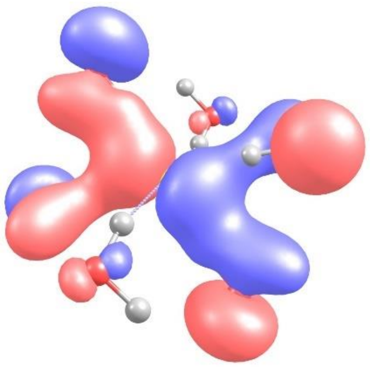

23

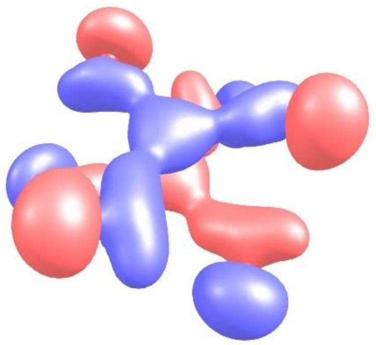

27

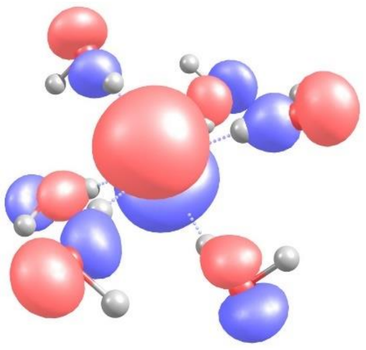

28

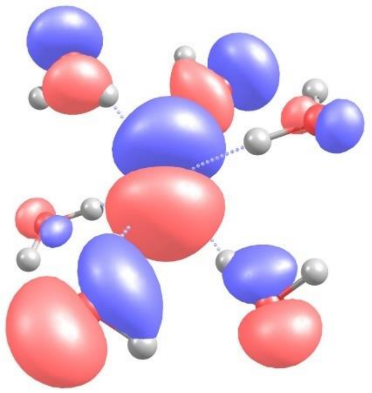

29

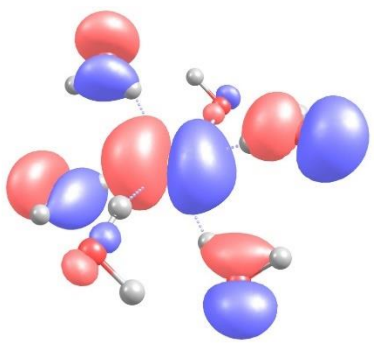

Figure S10. Structure of the $F^-(H_2O)_6$ cluster used for shielding analyses and isosurface plots (0.03 a.u.) of relevant occupied canonical molecular orbitals contributing to the ^{19}F shielding tensor.

Table S9. Computed ^{1}H NMR shifts of coordinating MeCN hydrogen atoms (CH…F interactions).[a]

anion	n	BHLYP	cLH12sir	cLH12sif	cLH20t
F^-	1	10.65	10.82	10.86	10.47
	2	8.55	8.69	8.70	8.45
	3	7.66	7.77	7.77	7.58
	4	6.90	6.98	6.98	6.86
	5	6.48	6.54	6.54	6.44
	6	6.50	6.54	6.53	6.46
	7	5.83	5.85	5.85	5.78
	8	5.19	5.21	5.20	5.16
	9	4.57	4.58	4.57	4.56
	10	5.15	5.17	5.16	5.12
	11	5.23	5.24	5.24	5.20
FHF^-	1	4.97	5.05	5.05	4.89
	2	4.75	4.82	4.81	4.68
	3	4.62	4.67	4.67	4.55
	4	4.63	4.67	4.67	4.59
	5	4.44	4.48	4.47	4.41
	6	4.42	4.45	4.44	4.39
	7	4.18	4.20	4.20	4.16
	8	4.15	4.17	4.16	4.12
	9	3.94	3.95	3.94	3.92
	10	3.98	3.98	3.97	3.96
	11	3.81	3.81	3.80	3.79
	12	3.50	3.49	3.48	3.48
ClF_2^-	1	3.83	3.90	3.90	3.79
	2	3.74	3.79	3.79	3.72
	3	3.64	3.69	3.69	3.62
	4	3.51	3.55	3.55	3.48
	5	3.43	3.47	3.46	3.40
	6	3.38	3.42	3.42	3.35
	7	3.34	3.38	3.37	3.31
	8	3.40	3.40	3.40	3.35
	9	3.54	3.56	3.56	3.52
	10	3.41	3.44	3.43	3.39
	11	3.36	3.36	3.35	3.33
	12	3.30	3.31	3.31	3.28
ClF_4^-	1	2.68	2.66	2.66	2.60
	2	2.65	2.63	2.63	2.57
	3	2.99	2.99	2.99	2.94
	4	3.12	3.14	3.14	3.10
	5	3.12	3.11	3.11	3.07
	6	3.16	3.16	3.16	3.11
	7	3.05	3.05	3.04	3.00
	8	2.97	2.96	2.95	2.92
	9	3.27	3.27	3.26	3.23
	10	3.02	3.01	3.00	2.98
	11	3.05	3.04	3.03	3.01
	12	2.92	2.90	2.90	2.90

[a]MARIJ-GIAO-DFT/pcSseg-3/COSMO//MARIJ-D3(BJ)-BP86/def2-TZVPP/COSMO level. Averaged over all n solvent molecules. The following absolute ^{1}H shieldings for the TMS reference standard were used: 31.44 ppm (BHLYP), 31.13 ppm (cLH12ct-SsirPW92), 31.07 ppm (cLH12ct-SsifPW92), 31.20 ppm (cLH20t). cLH12sir and cLH12sif abbreviate cLH12ct-SsirPW92 and cLH12ct-SsifPW92, respectively. The experimental ^{1}H-NMR shift of bulk MeCN is 1.96 ppm.[S4] The computed ^{1}H-NMR shifts of the isolated CH_3CN molecule at the chosen levels are 2.25 ppm (BHLYP), 2.18 ppm (cLH12ct-SsirPW92), 2.18 ppm (cLH12ct-SsifPW92), 2.16 ppm (cLH20t). Computed values for a small cluster $(CH3CN)_8$ are 2.65 ppm (BHLYP), 2.60 ppm (cLH12ct-SsirPW92), 2.60 ppm (cLH12ct-SsifPW92), 2.59 ppm (cLH20t). The "c" for the LH functionals denotes inclusion of current-density response.

S9. NMR shifts of coordinated and uncoordinated MeCN solvent molecules

Table S10. Computed ^{1}H NMR shifts of noncoordinating MeCN hydrogen atoms.[a]

anion	n	BHLYP	cLH12sir	cLH12sif	cLH20t
F^-	1	1.49	1.44	1.44	1.42
	2	1.75	1.69	1.69	1.69
	3	1.91	1.85	1.85	1.84
	4	1.98	1.92	1.92	1.91
	5	2.05	1.99	1.99	1.98
	6	1.96	1.90	1.90	1.90
	7	2.06	2.00	2.00	2.00
	8	2.15	2.09	2.09	2.09
	9	2.40	2.35	2.35	2.34
	10	2.37	2.32	2.32	2.31
	11	2.41	2.36	2.36	2.34
FHF^-	1	1.97	1.89	1.89	1.88
	2	2.09	2.01	2.01	2.01
	3	2.14	2.07	2.06	2.05
	4	2.19	2.12	2.12	2.12
	5	2.25	2.18	2.17	2.17
	6	2.24	2.17	2.17	2.18
	7	2.25	2.18	2.18	2.18
	8	2.28	2.21	2.21	2.22
	9	2.38	2.31	2.31	2.30
	10	2.36	2.30	2.30	2.30
	11	2.45	2.40	2.39	2.38
	12	2.49	2.43	2.43	2.43
ClF_2^-	1	2.16	2.07	2.07	2.06
	2	2.21	2.13	2.13	2.14
	3	2.36	2.29	2.29	2.28
	4	2.44	2.37	2.37	2.36
	5	2.43	2.36	2.36	2.35
	6	2.44	2.38	2.37	2.37
	7	2.54	2.47	2.47	2.46
	8	2.53	2.47	2.47	2.46
	9	2.55	2.49	2.49	2.48
	10	2.48	2.43	2.42	2.42
	11	2.50	2.45	2.44	2.44
	12	2.62	2.57	2.56	2.56
ClF_4^-	1	2.28	2.21	2.20	2.21
	2	2.28	2.20	2.20	2.20
	3	2.47	2.41	2.40	2.40
	4	2.37	2.30	2.30	2.29
	5	2.44	2.37	2.37	2.37
	6	2.42	2.35	2.34	2.34
	7	2.49	2.43	2.43	2.43
	8	2.49	2.44	2.43	2.43
	9	2.48	2.42	2.42	2.41
	10	2.57	2.51	2.50	2.50
	11	2.47	2.41	2.41	2.40
	12	2.61	2.55	2.55	2.54

[a] MARIJ-GIAO-DFT/pcSseg-3/COSMO//MARIJ-D3(BJ)-BP86/def2-TZVPP/COSMO level. Averaged over all n solvent molecules. The following absolute ^{1}H shieldings for the TMS reference standard were used: 31.44 ppm (BHLYP), 31.13 ppm (cLH12ct-SsirPW92), 31.07 ppm (cLH12ct-SsifPW92), 31.20 ppm (cLH20t). cLH12sir and cLH12sif abbreviate cLH12ct-SsirPW92 and cLH12ct-SsifPW92, respectively. The experimental ^{1}H-NMR shift of bulk MeCN is 1.96 ppm.[S4] The computed ^{1}H-NMR shifts of the isolated CH_3CN molecule at the chosen levels are 2.25 ppm (BHLYP), 2.18 ppm (cLH12ct-SsirPW92), 2.18 ppm (cLH12ct-SsifPW92), 2.16 ppm (cLH20t). Computed values for a small cluster $(CH3CN)_8$ are 2.65 ppm (BHLYP), 2.60 ppm (cLH12ct-SsirPW92), 2.60 ppm (cLH12ct-SsifPW92), 2.59 ppm (cLH20t). The “c” for the LH functionals denotes inclusion of current-density response.

Table S11. Computed MeCN methyl ^{13}C shifts.[a]

anion	n	BHLYP	cLH12sir	cLH12sif	cLH20t
F^-	1	-1.6	-0.7	-0.7	-0.2
	2	-0.1	0.8	0.8	1.1
	3	0.5	1.4	1.3	1.8
	4	0.9	1.8	1.7	2.0
	5	1.2	2.1	2.0	2.4
	6	1.2	2.1	2.0	2.2
	7	1.7	2.6	2.6	2.8
	8	2.4	3.3	3.3	3.6
	9	2.7	3.7	3.6	3.9
	10	2.9	3.8	3.7	4.2
	11	3.1	3.9	3.9	4.3
FHF^-	1	-0.4	0.5	0.4	0.8
	2	0.2	1.0	1.0	1.4
	3	0.4	1.2	1.2	1.6
	4	1.2	2.1	2.1	2.3
	5	1.8	2.6	2.6	3.0
	6	2.1	2.9	2.8	3.2
	7	2.4	3.1	3.1	3.4
	8	2.3	3.1	3.0	3.4
	9	2.5	3.2	3.2	3.6
	10	3.3	4.1	4.0	4.4
	11	3.2	4.0	3.9	4.4
	12	3.7	4.5	4.4	4.9
ClF_2^-	1	-0.2	0.7	0.6	1.0
	2	0.9	1.7	1.7	1.8
	3	1.4	2.2	2.1	2.6
	4	1.6	2.4	2.3	2.7
	5	1.8	2.6	2.6	3.0
	6	2.2	3.0	2.9	3.4
	7	1.6	2.4	2.3	2.8
	8	2.9	3.7	3.6	4.1
	9	3.2	3.9	3.8	4.3
	10	3.6	4.3	4.2	4.7
	11	3.6	4.3	4.3	4.8
	12	3.5	4.3	4.2	4.8
ClF_4^-	1	1.0	1.7	1.7	2.0
	2	1.1	1.8	1.7	2.0
	3	2.2	2.9	2.8	3.3
	4	2.9	3.6	3.5	4.0
	5	2.3	3.1	3.1	3.5
	6	3.6	4.3	4.2	4.8
	7	3.0	3.7	3.6	4.2
	8	3.1	3.8	3.7	4.3
	9	3.1	3.9	3.8	4.3
	10	3.9	4.7	4.6	5.2
	11	4.0	4.7	4.6	5.2
	12	3.9	4.5	4.5	4.9

[a]MARIJ-GIAO-DFT/pcSseg-3/COSMO//MARIJ-D3(BJ)-BP86/def2-TZVPP/COSMO level. Averaged over all n solvent molecules. The following ^{13}C shieldings for the TMS reference were used: 184.1 ppm (BHLYP), 192.3 ppm (cLH12ct-SsirPW92), 193.8 ppm (cLH12ct-SsifPW92), 190.6 ppm (cLH20t). cLH12sir and cLH12sif abbreviate cLH12ct-SsirPW92 and cLH12ct-SsifPW92. The experimental ^{13}C-NMR shift of bulk MeCN is 1.79 ppm.[S4] The computed ^{13}C-NMR shifts at the chosen levels of an isolated CH_3CN molecule are 0.2 ppm (BHLYP), 0.9 ppm (cLH12ct-SsirPW92), 0.8 ppm (cLH12ct-SsifPW92), 1.2 ppm (cLH20t), those of a small cluster $(CH_3CN)_8$ are 3.8 ppm (BHLYP), 4.4 ppm (cLH12ct-SsirPW92), 4.4 ppm (cLH12ct-SsifPW92), 4.8 ppm (cLH20t). The "c" for the LH functionals denotes inclusion of current-density response.

Table S12. Computed MeCN nitrile ^{13}C shifts.[a]

anion	n	BHLYP	cLH12sir	cLH12sif	cLH20t
F^-	1	142.7	141.9	142.4	141.3
	2	141.2	140.4	140.9	139.8
	3	140.6	139.8	140.4	139.1
	4	140.2	139.4	139.9	138.7
	5	139.9	139.1	139.6	138.5
	6	139.7	138.9	139.4	138.3
	7	139.0	138.3	138.8	137.6
	8	138.5	137.7	138.3	137.0
	9	138.4	137.7	138.2	136.9
	10	138.9	138.1	138.7	137.4
	11	139.1	138.3	138.8	137.6
FHF^-	1	139.1	138.6	139.1	137.8
	2	139.3	138.7	139.2	137.9
	3	139.3	138.7	139.2	137.9
	4	138.8	138.1	138.6	137.4
	5	139.0	138.2	138.8	137.6
	6	138.6	137.9	138.4	137.2
	7	138.5	137.7	138.3	137.1
	8	138.6	137.8	138.4	137.2
	9	138.6	137.8	138.3	137.2
	10	138.6	137.8	138.3	137.1
	11	138.7	137.9	138.4	137.2
	12	138.2	137.5	138.0	136.8
ClF_2^-	1	138.6	138.0	138.5	137.2
	2	138.4	137.8	138.3	137.1
	3	139.3	138.5	139.0	137.8
	4	139.2	138.5	139.0	137.7
	5	138.8	138.1	138.6	137.3
	6	138.9	138.1	138.7	137.4
	7	139.2	138.5	139.0	137.8
	8	138.8	138.0	138.6	137.4
	9	139.0	138.2	138.8	137.5
	10	138.7	137.9	138.4	137.2
	11	138.7	137.9	138.4	137.2
	12	138.8	138.0	138.5	137.3
ClF_4^-	1	137.8	137.3	137.9	136.3
	2	137.7	137.3	137.8	136.3
	3	138.4	137.8	138.4	137.0
	4	138.5	137.8	138.3	137.1
	5	138.4	137.7	138.2	137.1
	6	138.8	138.0	138.5	137.4
	7	138.9	138.2	138.7	137.5
	8	138.2	137.5	138.0	136.8
	9	138.7	137.9	138.5	137.2
	10	138.6	137.8	138.3	137.2
	11	138.8	138.0	138.5	137.3
	12	138.4	137.7	138.2	137.0

[a]MARIJ-GIAO-DFT/pcSseg-3/COSMO//MARIJ-D3(BJ)-BP86/def2-TZVPP/COSMO level. Averaged over all n solvent molecules. The following ^{13}C shieldings for the TMS reference were used: 184.1 ppm (BHLYP), 192.3 ppm (cLH12ct-SsirPW92), 193.8 ppm (cLH12ct-SsifPW92), 190.6 ppm (cLH20t). cLH12sir and cLH12sif abbreviate cLH12ct-SsirPW92 and cLH12ct-SsifPW92. The experimental nitrile ^{13}C-NMR shift is 118.26 ppm.[S4] The computed ^{13}C-NMR shifts at the chosen levels for an isolated CH_3CN molecule are 137.2 ppm (BHLYP), 136.6 ppm (cLH12ct-SsirPW92), 137.1 ppm (cLH12ct-SsifPW92), 135.8 ppm (cLH20t), those for a small cluster $(CH_3CN)_8$ are 139.7 ppm (BHLYP), 138.7 ppm (cLH12ct-SsirPW92), 139.3 ppm (cLH12ct-SsifPW92), 138.2 ppm (cLH20t). The "c" for the LH functionals denotes inclusion of current-density response.

Table S13. Computed MeCN ^{15}N shifts.[a]

anion	n	BHLYP	cLH12sir	cLH12sif	cLH20t
F^-	1	-136.0	-148.4	-148.5	-145.3
	2	-131.8	-144.3	-144.4	-141.5
	3	-129.2	-141.7	-141.8	-138.5
	4	-128.0	-140.6	-140.7	-137.4
	5	-127.0	-139.5	-139.7	-136.6
	6	-127.8	-140.2	-140.3	-137.5
	7	-125.9	-138.4	-138.6	-135.6
	8	-123.5	-136.2	-136.3	-133.2
	9	-120.7	-133.6	-133.8	-130.2
	10	-122.5	-135.0	-135.1	-132.0
	11	-121.8	-134.3	-134.5	-131.3
FHF^-	1	-130.5	-143.0	-143.1	-140.1
	2	-129.0	-141.6	-141.7	-138.6
	3	-128.4	-141.0	-141.1	-137.9
	4	-125.1	-137.7	-137.8	-134.8
	5	-124.8	-137.3	-137.5	-134.5
	6	-123.8	-136.4	-136.5	-133.4
	7	-123.4	-136.0	-136.1	-133.1
	8	-123.5	-136.1	-136.2	-133.2
	9	-122.3	-134.9	-135.0	-132.1
	10	-122.4	-134.9	-135.0	-132.1
	11	-121.8	-134.2	-134.4	-131.4
	12	-120.5	-133.0	-133.2	-130.2
ClF_2^-	1	-129.5	-142.0	-142.2	-139.1
	2	-126.0	-138.7	-138.8	-136.0
	3	-125.5	-138.0	-138.1	-134.9
	4	-125.1	-137.7	-137.8	-134.5
	5	-124.5	-137.1	-137.2	-134.2
	6	-123.9	-136.5	-136.6	-133.4
	7	-124.1	-136.8	-136.9	-133.7
	8	-123.1	-135.6	-135.7	-132.8
	9	-122.8	-135.3	-135.4	-132.2
	10	-122.4	-134.8	-135.0	-131.9
	11	-122.1	-134.5	-134.6	-131.7
	12	-121.6	-134.0	-134.1	-131.2
ClF_4^-	1	-128.2	-141.0	-141.1	-137.8
	2	-128.1	-140.9	-141.0	-137.7
	3	-125.8	-138.6	-138.7	-135.6
	4	-124.7	-137.2	-137.3	-134.3
	5	-123.4	-136.0	-136.1	-133.3
	6	-123.4	-135.7	-135.9	-133.1
	7	-123.8	-136.3	-136.4	-133.4
	8	-122.4	-135.0	-135.1	-132.1
	9	-123.3	-135.7	-135.8	-132.8
	10	-121.6	-134.0	-134.1	-131.2
	11	-122.8	-135.0	-135.1	-132.1
	12	-121.5	-133.9	-134.0	-131.1

[a]MARIJ-GIAO-DFT/pcSseg-3/COSMO//MARIJ-D3(BJ)-BP86/def2-TZVPP/COSMO level. Averaged over all n solvent molecules. Shifts relative to NH_3 (GP): 256.9 ppm (BHLYP), 267.1 ppm (cLH12ct-SsirPW92), 268.6 ppm (cLH12ct-SsifPW92), 265.0 ppm (cLH20t) using a gas to liquid shift ($\sigma_{liq.}$- σ_{gas}; T:298-350K) of -19.47 ppm for NH_3 (Ref. S5), and a secondary shift of -380.2 ppm (Ref. S6); cLH12sir and cLH12sif correspond to cLH12ct-SsirPW92 and cLH12ct-SsifPW92. The experimental ^{15}N-NMR shift of bulk MeCN is 136.4 ppm.[S6] The computed ^{15}N-NMR shifts at the chosen levels of an isolated CH_3CN molecule are -127.1 ppm (BHLYP), -139.6 ppm (cLH12ct-SsirPW92), -139.7 ppm (cLH12ct-SsifPW92), -136.9 ppm (cLH20t), those of a small cluster $(CH_3CN)_8$ are -119.5 ppm (BHLYP), -131.6 ppm (cLH12ct-SsirPW92), -131.7 ppm (cLH12ct-SsifPW92), -128.6 ppm (cLH20t). The "c" for the LH functionals denotes inclusion of current-density response.

References

S1. M. Gerken, J. A. Boatz, A. Kornath, R. Haiges, S. Schneider, T. Schroer, K. O. Christe, *J. Fluor. Chem.* **2002**, *116*, 49-58.

S2. K. O. Christe, W. W. Wilson, *J. Fluor. Chem.* **1990**, *47*, 117-120.

S3. P. Pröhm, J. R. Schmid, K. Sonnenberg, P. Voßnacker, S. Steinhauer, C. J. Schattenberg, R. Müller, M. Kaupp, S. Riedel, *Angew. Chem. Int. Ed. Engl.* **2020**, *59*, 16002-16006.

S4. H. E. Gottlieb, V. Kotlyar, A. Nudelman, *J. Org. Chem.* **1997**, *62*, 7512-7515.

S5. C. J. Jameson, A. K. Jameson, S. M. Cohen, H. Parker, D. Oppusunggu, P. M. Burrell, S. Wille, *J. Chem. Phys.* **1981**, *74*, 1608-1612.

S6. C. J. Jameson, A. K. Jameson, D. Oppusunggu, S. Wille, P. M. Burrell, J. Mason, *J. Chem. Phys.* **1981**, *74*, 81-88.
